# Genome organization and genomics in *Chlamydia*: whole genome sequencing increases understanding of chlamydial virulence, evolution, and phylogeny

**DOI:** 10.3389/fcimb.2023.1178736

**Published:** 2023-05-23

**Authors:** Laurence Don Wai Luu, Vasilli Kasimov, Samuel Phillips, Garry S. A. Myers, Martina Jelocnik

**Affiliations:** ^1^ School of Life Sciences, University of Technology Sydney, Sydney, NSW, Australia; ^2^ Centre for Bioinnovation, University of the Sunshine Coast, Sippy Downs, QLD, Australia; ^3^ School of Science, Technology and Engineering, University of the Sunshine Coast, Sippy Downs, QLD, Australia; ^4^ Australian Institute for Microbiology and Infection, University of Technology Sydney, Sydney, NSW, Australia

**Keywords:** *Chlamydiaceae*, *Chlamydia trachomatis*, genomics, genome content, whole genome sequence, next generation sequencing, tissue tropism, host tropism

## Abstract

The genus *Chlamydia* contains important obligate intracellular bacterial pathogens to humans and animals, including *C. trachomatis* and *C. pneumoniae*. Since 1998, when the first *Chlamydia* genome was published, our understanding of how these microbes interact, evolved and adapted to different intracellular host environments has been transformed due to the expansion of chlamydial genomes. This review explores the current state of knowledge in *Chlamydia* genomics and how whole genome sequencing has revolutionised our understanding of *Chlamydia* virulence, evolution, and phylogeny over the past two and a half decades. This review will also highlight developments in multi-omics and other approaches that have complemented whole genome sequencing to advance knowledge of *Chlamydia* pathogenesis and future directions for chlamydial genomics.

## 1 Introduction

**Figure 1:**
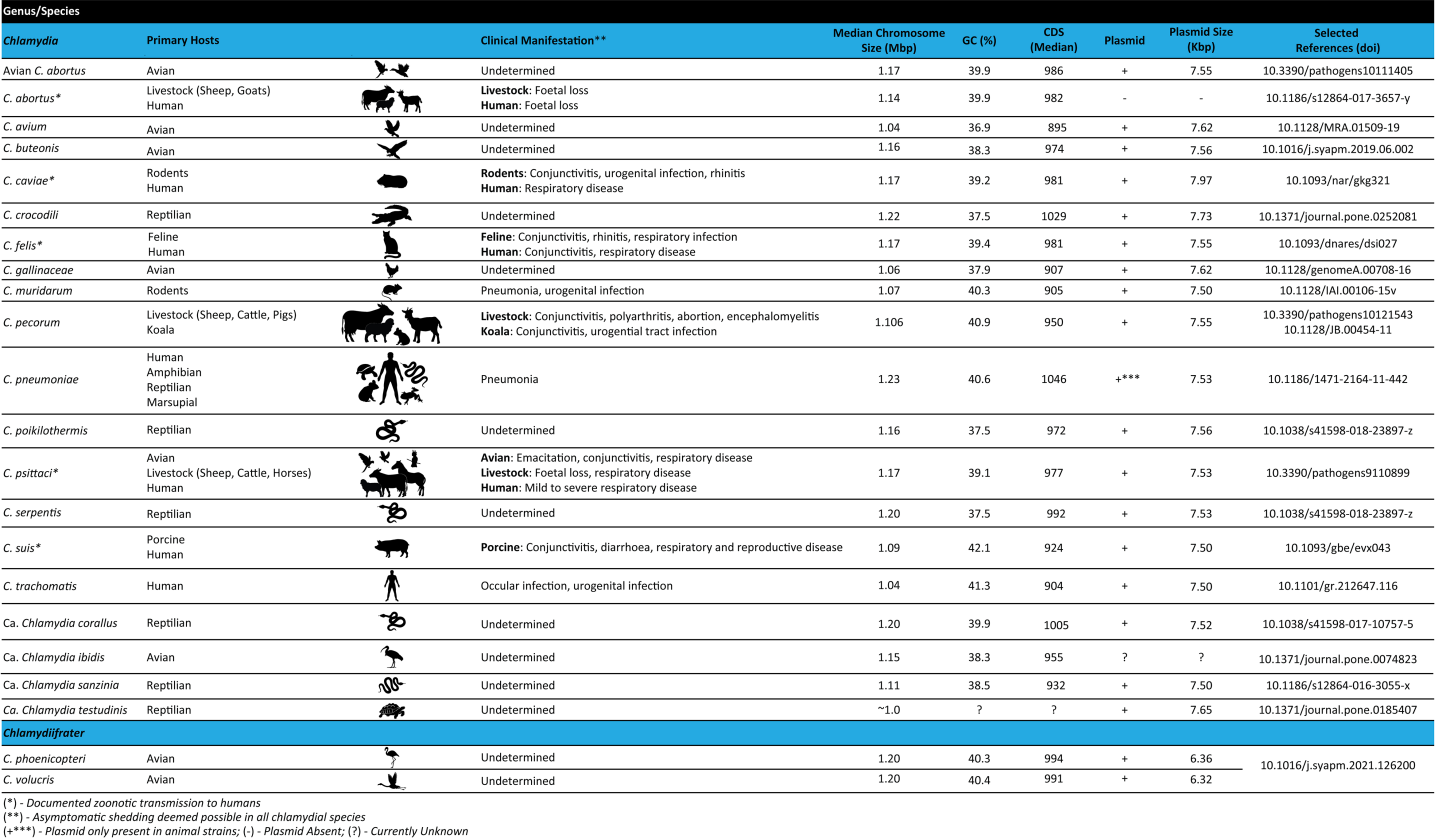
Features of the *Chlamydiaceae* family including primary host range, clinical manifestations and genome characteristics.

Within the last 15 years, advances in whole genome sequencing (WGS) technologies and expansions in publicly available chlamydial genomes have dramatically increased our knowledge on chlamydial evolution and phylogeny, genome structure, metabolic processes, and potential virulence factors of these organisms. Presently, WGS is considered the gold standard for molecular epidemiology, evolution, and genetic diversity, and has become an integral part of cell biology studies in the genetically recalcitrant *Chlamydia*. The distinguishing feature of *Chlamydia* is its biphasic developmental cycle which alternates between extracellular infectious elementary bodies (EBs), which attach to and enter the host cell, and intracellular replicative reticulate bodies (RBs) residing in a membrane-enclosed inclusion. Upon RBs replication and inclusion growth, they dedifferentiate into EBs, to be released out of host cells and continue the infectious process (Elwell et al., 2016; Gitsels et al., 2019; Chiarelli et al., 2020). Under stress, *Chlamydia* RBs can also dedifferentiate into a non-replicating, persistent form, known as aberrant bodies (ABs) (Panzetta et al., 2018). The chlamydial lifecycle is central to the pathogenesis of these bacteria, as their survival depends on the complex host-pathogen interactions to establish an intracellular niche, subvert host cellular processes, acquire host-derived nutrients and evade the host immune response (Elwell et al., 2016; Panzetta et al., 2018; Gitsels et al., 2019; Chiarelli et al., 2020). Currently, the *Chlamydiaceae* consists of two genera: 1) *Chlamydia* (**Figure 1**), which harbours the following characterised species: *C. abortus*, *C. avium*, *C. buteonis*, *C. caviae*, *C. crocodili*, *C. felis*, *C. gallinacea*, *C. muridarum*, *C. pecorum*, *C. pneumoniae*, *C. poikilothermis*, *C. psittaci*, *C. serpentis*, *C. suis*, *C. trachomatis*, in addition to four Candidatus (Ca.) species: Ca. *Chlamydia corallus*, Ca. *Chlamydia ibidis*, Ca. *Chlamydia sanzinia* and Ca. *Chlamydia testudines* (Cheong et al., 2019; Zaręba-Marchewka et al., 2021) and, 2) the newly described *Chlamydiifrater* (**Figure 1**), harbouring the two recent species, *C. phoenicopteri* and *C. volucris* (Vorimore et al., 2021b). The availability of genome sequences for all novel and well-characterised species within the *Chlamydiaceae* provides an unparalleled opportunity to advance our understanding of the genetic diversity and taxonomy, genome biology, and gene content of these fascinating organisms (**Figure 1**). This review provides an overview of WGS methods and the most common genomic analyses, followed by a description of the chlamydial genome structure and content. It also provides details on the currently known events of genomic recombination as it relates to the threat of genetic transfer of antibiotic resistance genes, and challenges to the identification of lymphogranuloma venereum (LGV) outbreaks. Finally, we conclude with a discussion of anticipated directions for chlamydial genomics.

## 2 Chlamydial genomics: A new gold standard for chlamydial studies

### 2.1 WGS and genomic analyses

For most bacterial species, obtaining high-yield DNA extracted from cultured organisms remains the gold standard for WGS. Due to the intracellular niche of *Chlamydiaceae*, culturing and isolation is laborious, generally yielding lower levels of chlamydial genetic material contaminated with host DNA. In the past decade, there have been advances in obtaining chlamydial genomes from contaminated and/or low-yielding DNA samples retrieved from clinical and environmental samples using non-targeted and targeted approaches. These developments have resulted in a substantial increase of WGS data being generated (Bachmann et al., 2014; Taylor-Brown et al., 2018). New targeted WGS approaches are preferred for epidemiological and diagnostic purposes, particularly if the organism has already been sequenced, due to lower costs, higher throughput, and increased specificity (Taylor-Brown et al., 2018). However, for chlamydial discovery (e.g. novel species), non-targeted approaches are mostly utilised. Other methods also include expensive deep sequencing of contaminated samples using bioinformatic filtering to determine chlamydial sequences.

For WGS studies using (high-yield) DNA extracted from cultured isolates, direct next generation sequencing (NGS) is commonly used. Due to decreasing costs and ongoing developments in NGS, hybrid approaches such as nanopore sequencing to complement short-read WGS, are used to improve and/or provide closed complete genomes for *Chlamydia* (Heijne et al., 2021; Zaręba-Marchewka et al., 2021). In contrast, WGS of *Chlamydia*-specific and/or as a part of metagenomic sequencing from clinical swabs often yield poor results. However, this limitation was overcome with the development of culture-independent sample preparation methods, further supplemented with targeted capture methods (Joseph et al., 2015; Taylor-Brown et al., 2018; Zaręba-Marchewka et al., 2020). One of these methods is probe-based sequence-specific capture using biotinylated 120mer RNA probes called baits (Christiansen et al., 2014). This approach was successfully performed on WGS of *C. trachomatis* DNA from clinical samples (Christiansen et al., 2014; Hadfield et al., 2017; Seth-Smith et al., 2021), livestock and koala *C. pecorum* DNA from mucosal animal samples (Bachmann et al., 2015), DNA from archival *C. pneumoniae* strains (Roulis et al., 2015a; Roulis et al., 2015b), as well as *C. psittaci* clinical isolates (White et al., 2022) and DNA samples form a range of hosts (Branley et al., 2016). The recent development of third generation long-read sequencing technologies such as Oxford Nanopore and PacBio has already been used to provide high-quality complete *Chlamydia* genomes, resolving complex repetitive and recombination regions (Livingstone et al., 2021; Zaręba-Marchewka et al., 2021). These technologies also contain the additional potential for understanding chlamydial biology which is yet to be fully understood. For instance, Nanopore sequencing can directly detect DNA and RNA methylation in bacteria such as 6mA through changes in voltage signals when modified bases pass through the nanopore (Tourancheau et al., 2021), while PacBio Hi-C sequencing can be used to reveal the 3D genome structure and chromatin interactions (Lamy-Besnier et al., 2021). These novel technologies can be used to explore epigenetic modifications and 3D genome structure in different *Chlamydia* species, strains and/or stages of infection.

With the decreasing costs of deep sequencing, improvements in host DNA depletion techniques and advances in bioinformatic algorithms, shotgun metagenome sequencing which involves direct, untargeted sequencing of all microbial DNA in samples without the need for culture is becoming increasingly common. Shotgun metagenome sequencing provides new opportunities to study both *Chlamydia* and the microbiome; the microbiome has been shown to impact *Chlamydia* colonisation (Edwards et al., 2019). The successful recovery of metagenome-assembled genomes (MAGS) from shotgun metagenome data can also be used to discover new genera or species, as illustrated by the discovery of novel chlamydial lineages in anoxic marine sediments (Dharamshi et al., 2020).

Following WGS, analysis of *Chlamydia* genomes mainly follows a straightforward approach including *de novo* assembly into contigs and mapping approaches. Assembled sequences are subjected to genome annotation and estimation of genomic variability using single nucleotide polymorphisms (SNPs) and comparative approaches. Finally, a variety of phylogenomic analyses are performed to establish phylogenetic relationships among analysed strains and/or species (Joseph et al., 2015; Wolff et al., 2015; Hadfield et al., 2017; Sigalova et al., 2019; Hölzer et al., 2020; White et al., 2021; Zaręba-Marchewka et al., 2021; White et al., 2022). The reads and/or assembled genomes are deposited in publicly available databases so that they can be re-used across studies, providing a valuable resource for the chlamydial research community.

In chlamydial genomic studies, *in silico* analyses elucidating virulence factors and/or gene functions to explain phenotypes are also common. However, genes encoding for hypothetical or unknown proteins account for a significant proportion (39 – 45%) of most chlamydial genomes. Such hypothetical protein genes are conserved and considered “chlamydial-specific hypothetical protein genes”, with homologues commonly found among chlamydial species (Sigalova et al., 2019; Hölzer et al., 2020). Despite major advances in the genetic manipulation of chlamydiae, the function of many chlamydial virulence, tissue tropic-, and metabolic factors remain unknown (Bastidas and Valdivia, 2016). WGS and comparative genomics are efficient ways to characterise the genome content for strains of interest, further enabling detailed investigation of specific genotypes and delineating factors for host specificity, pathogenicity and tissue tropisms. One method for determining gene function is querying predicted Open Reading Frame (ORF) or coding DNA sequences (CDS) and assigning gene functions against a reference protein database (e.g. UniProtKB or Pfam-A database). Further gene and protein sequence homology or conserved domain analyses are commonly performed in chlamydial WGS annotation studies utilising publicly available or custom databases (Voigt et al., 2012; Read et al., 2013; Borges and Gomes, 2015; Seth-Smith et al., 2017a; Borges et al., 2019; Sigalova et al., 2019; Hölzer et al., 2020; Heijne et al., 2021; White et al., 2021). Recently, Pillonel and colleagues developed “open-access ChlamDB”, a comparative genomics database containing 277 genomes covering the entire *Chlamydiae* phylum as well as their closest relatives belonging to the *Planctomycetes-Verrucomicrobiae*-*Chlamydiae* (PVC) superphylum. Open-access ChlamDB provides various tools for comparing, analysing and retrieving *Chlamydiae*-specific genomic data (Pillonel et al., 2020). Such *in silico* analyses enable predictions of certain phenotypic or pathogenicity traits of chlamydial organisms. However, these require experimental validation, with limitations depending on the computational methods and resources used (Zhao et al., 2020). For genes encoding hypothetical proteins, comparative genomics to reveal conserved or absent genes in different lineages or tropic strains can provide valuable insight into their potential function and help identify genes for further investigation. Finally, using WGS in phylogenetic analyses can provide fine-detailed phylogeny, epidemiological networks and evolutionary pathways of *Chlamydia* species by estimating the chlamydial molecular clock. Without WGS techniques, this level of resolution would not be achievable, and certain evolutionary important lineages could be missed.

## 3 Chlamydial genome organisation and content: variability within similarity and synteny

**Figure 2:**
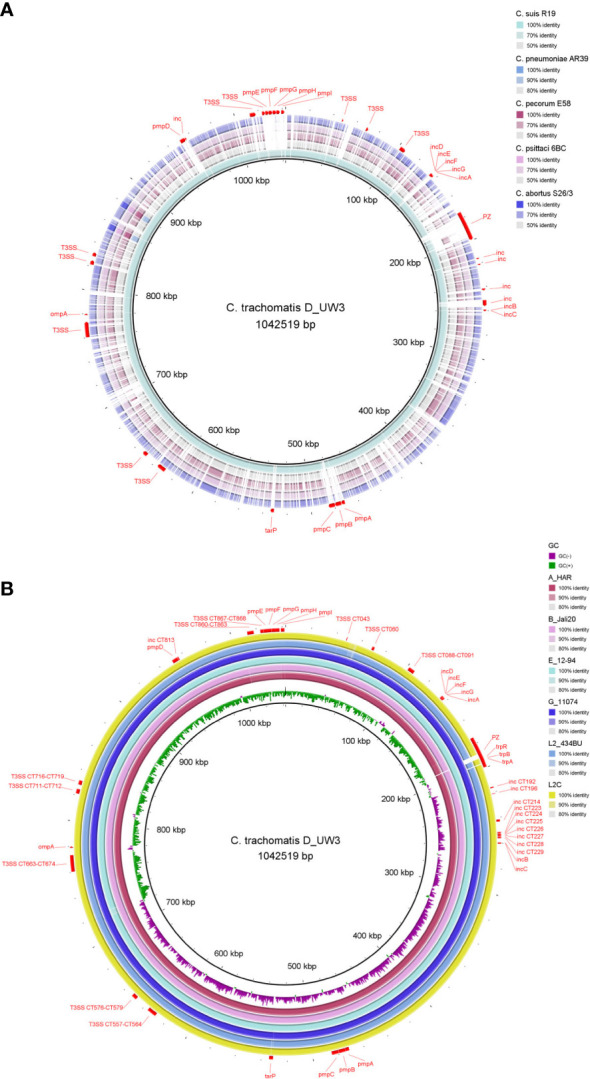
Chromosome comparison of selected genomes from **(A)** major human and animal *Chlamydia* pathogens (*C. trachomatis, C suis, C pneumoniae, C pecorum, C psittaci* and *C abortus*) and **(B)** *C trachomatis* strains from the three main lineages: ocular (A_HAR, B_Jali20), urogenital (D_UW3, E_12-94, G_11074) and LGV (L2_434BU, L2C). The *C trachomatis* strain D_UW3 was used as the reference. Important genome elements are annotated in red including the plasticity zone (PZ), *omp*A, *pmps*, *Incs*, T3SS, *tarP* and *trp* operon. The figure was generated using BRIG (v 0.95).

Genomes of *Chlamydiaceae* are compact, remarkably conserved and syntenic. Similar to most other obligate intracellular bacteria, chlamydial genomes have a significantly reduced, circularised chromosome at approximately 1-1.2 Mbp with 900 - 1500 CDSs (**Figures 1**, **2**) (Collingro et al., 2011; Voigt et al., 2012; Joseph et al., 2015; Pillonel et al., 2018; Sigalova et al., 2019). Almost all *Chlamydiaceae* species contain a highly conserved chlamydial plasmid of approximately 6.3 - 8 kbp, apart from livestock *C. abortus* strains, human strains of *C. pneumoniae*, and rare plasmid-free strains (Zhong, 2017; Szabo et al., 2020). The reduced genome for *Chlamydiaceae* species is a result of genome streamlining rather than degradation and is thought to be due to their transition to the intracellular lifestyle and co-evolution within their eukaryotic hosts (Moran, 2002; Collingro et al., 2011; Nunes and Gomes, 2014; Dharamshi et al., 2023). Within the same chlamydial species, the genomes are highly syntenic and similar, with a sequence homology of approximately 90% (**Figure 2**). Most of the differences are attributed to SNPs which have been postulated to play a role in virulence, host, and tissue tropism (Nunes et al., 2013; Read et al., 2013; Bachmann et al., 2014; Bachmann et al., 2015; Wolff et al., 2015).

Almost all *Chlamydia* spp. genomes possess several common well-characterised metabolic, species-specific, and virulence genes or genomic regions. Recent pan-genomic analyses of 227 *Chlamydia* genomes demonstrated the degree of chlamydial similarity, with over 81% of genes universally (~700 universal genes) or partially conserved (~967 periphery genes), and only 19% of genes (~380 genes) unique to singular genomes. According to homology analyses, the periphery genes within species accounted for differences in organic molecule transport and metabolism, while unique genes contributed to differences in intracellular trafficking, secretion, and vesicular transport (Sigalova et al., 2019). Due to their host-dependency and reduced genome size, *Chlamydiaceae* lack genes encoding essential metabolic enzymes, requiring them to siphon amino acids, nucleotides, and cofactors from the infected host cell. This symbiotic relationship results in phenotypic variations involving tissue and host tropisms among different *Chlamydiaceae* species. The estimated number of pseudogenes in each genome is low possibly due to genome streamlining and a strong selection pressure to remove non-essential genes (Sigalova et al., 2019). The estimated number of pseudogenes ranges from 5 - 8 in *C. pecorum*, 9 - 12 in *C. trachomatis*, *C. muridarum*, and *C. suis* to 21 in *C. pneumoniae* and 28 - 29 in *C. abortus* and *C. psittaci*, with premature stop codons commonly occurring in genes encoding hypothetical proteins, and virulence-associated genes (*pmps*, *Incs*, and cytotoxin) (Voigt et al., 2012; Bachmann et al., 2014; Borges and Gomes, 2015; Sigalova et al., 2019; White et al., 2021).

**Figure 3:**
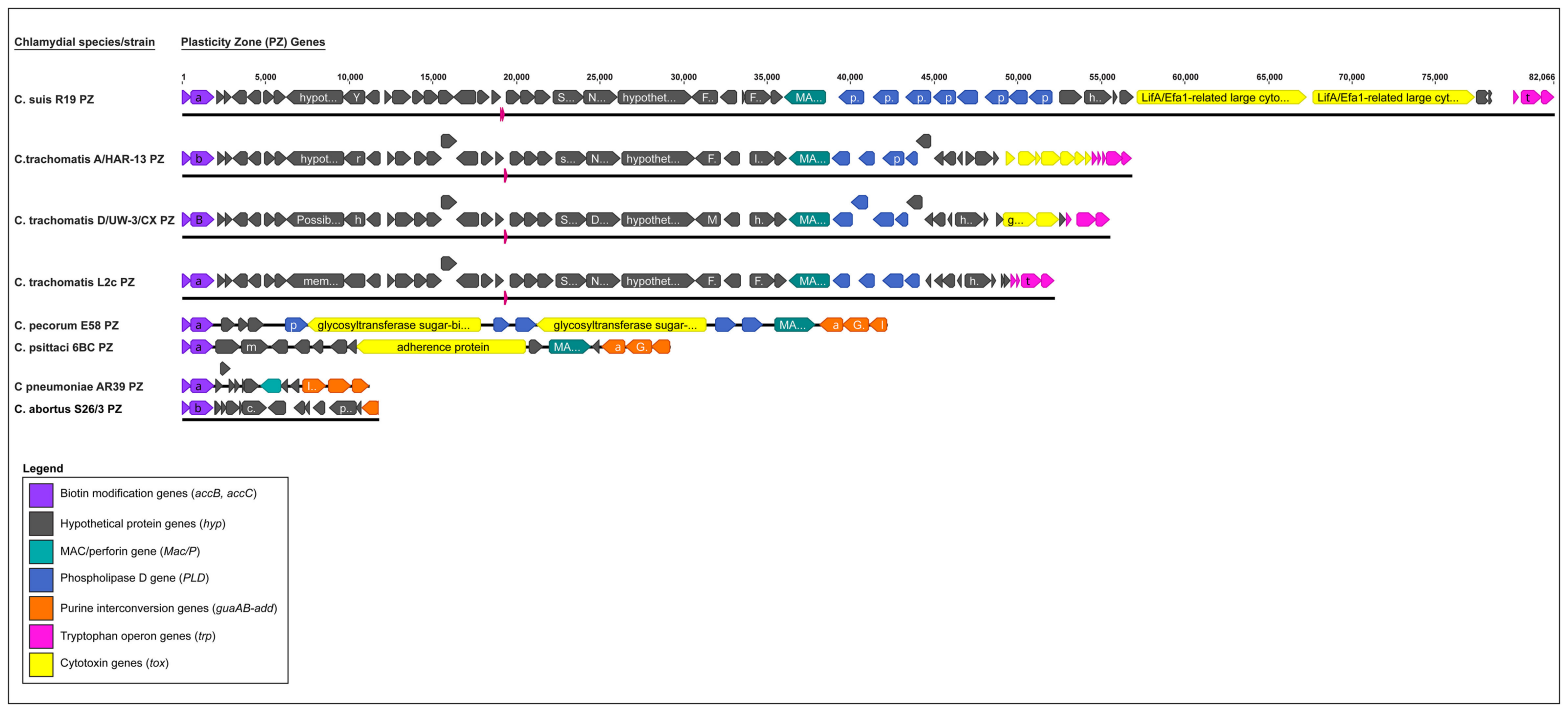
Comparison of the plasticity zone (PZ) from selected major human and animal *Chlamydia* species (*C. suis, C. trachomatis, C. pecorum, C. psittaci, C. pneumoniae* and *C. abortus*). The PZ elements are depicted in different colours, where biotin modification genes (*acc*B, *acc*C) are in purple, hypothetical protein (*hyp*) genes are in grey, MAC/perforin (*MAC/P*) in teal, Phospholipase D (*PL*D) in blue, purine interconversion genes (*gua*AB, *add*) in orange, Tryptophan operon genes (*trp*) in pink, and chlamydial cytotoxin (*tox*) gene(s) in yellow.

Despite a high level of genome conservation, *Chlamydiaceae* possess several distinct polymorphic regions (**Figure 2**), with the highest variation occurring within the plasticity zone (PZ) (**Figure 3**). Other highly variable elements include genes encoding polymorphic membrane (*pmp*s) and inclusion membrane proteins (*Inc*s), Type III secretion system (T3SS), and other metabolic pathways, such as tryptophan biosynthesis (Bachmann et al., 2014; Sigalova et al., 2019; Hölzer et al., 2020) (**Figure 2**). Furthermore, *Chlamydiaceae* have been shown to possess strong recombination potential, particularly within virulence-associated genes, including the major outer membrane protein (*omp*A), T3SS effector translocated actin-recruiting protein (*tarP*), *pmp* genes and the PZ (Harris et al., 2012; Read et al., 2013; Joseph et al., 2015; Borges et al., 2019; White et al., 2021).

### 3.1 Tissue-tropic and virulence genes revealed by WGS

#### 3.1.1 The plasticity zone

An evolutionary conserved but highly variable genomic region, the PZ is a genomic region associated with bacterial virulence protein genes and is responsible for distinct niche characteristics specific to different *Chlamydia* species (Rajaram et al., 2015; Dimond et al., 2021). Among the pathogenically diverse *Chlamydia* spp., the PZ ranges from ∼5 kb for *C. avium* (five genes), ~20 – 40 kb for *C. abortus*, *C. psittaci* and *C. pecorum*, (~15 to 20 genes), to ~55 kb and ~81 kb for *C. trachomatis* and *C. muridarum* (> 45 genes) (**Figure 3**) (Hölzer et al., 2020; Dimond et al., 2021; Zaręba-Marchewka et al., 2021). This region commonly includes homologs to biotin modification genes *accB* and *accC*, MAC/perforin (*MacP*), a variable number of phospholipase D-like genes (*PLD*), cytotoxin (*tox*) homologs, purine interconversion genes *guaA* and *guaB*, and a different subset of species-specific hypothetical proteins. Additionally, a near-full tryptophan (*Trp)* operon can be found in the PZ regions of *C. caviae* and *C. felis*, while in *C. trachomatis* and *C. suis*, the PZ regions only contain partial *Trp* operons (Bachmann et al., 2014; Rajaram et al., 2015; Zaręba-Marchewka et al., 2021).

Of these PZ elements, only *accB* and *accC* are common to all *Chlamydia* and are considered the 5’ boundary of the PZ, while other PZ genes vary between the species. Notably, chlamydial *tox* genes have significant homology to the large clostridial toxins (LCTs) and are present in almost all *Chlamydia* spp., however it may vary in copy number. *C. muridarum* has three *tox* copies, *C. pecorum* and *C. suis* two copies, while *C. trachomatis*, *C. caviae, C. crocodile, C. felis*, *C. gallinacea*, *C. psittaci*, *C. buteonis*, and avian *C. abortus* strains have one *tox* gene. A cytotoxin gene is absent from the PZs of the human pathogen *C. pneumoniae* and the ovine pathogen *C. abortus* (**Figure 3**) (Bachmann et al., 2014; Heijne et al., 2021; Zaręba-Marchewka et al., 2021). Significantly, *tox* gene polymorphisms have been useful in defining *C. trachomatis* disease phenotypes where the *tox* ORFs can be intact in urogenitotropic strains, truncated in oculotropic strains, or absent altogether in the LGV invasive strains (**Figures 2B**, **3**) (Carlson et al., 2004; Nunes et al., 2013; Bachmann et al., 2014). Some PZ ORFs (e.g. species-specific hypothetical proteins) have no homologs or have been classified into families based on the nucleotide and/or protein sequence homology.

#### 3.1.2 Type 3 secretion system genes

All *Chlamydia* spp. possess conserved genes encoding for structural, effector and chaperone proteins of the evolutionary conserved T3SS, essential for the translocation of effector proteins directly into host cells. The secreted T3SS effectors are recognised as virulence factors in *Chlamydiae* (Peters et al., 2007; Bachmann et al., 2014; Mueller et al., 2014; Mojica et al., 2015). While for some species, like *C. trachomatis* and *C. psittaci*, T3SS effectors have been experimentally confirmed and/or functionally characterised (Mojica et al., 2015; da Cunha et al., 2017; Marschall et al., 2020). For other less characterised chlamydial species WGS analyses is used to identify and decipher their roles (Hölzer et al., 2020; Heijne et al., 2021; White et al., 2021).

In *Chlamydia* spp. genomes, T3SS genes consist of 20 – 25 genes, encoding for highly conserved T3SS apparatus scaffolding (e.g. chlamydial *Cds*C, *Cds*J, and *Cds*D homologs), inner membrane components (e.g. *Cds* R/S/T/U/V and *Cds* L/N/Q), extracellular needles (e.g. *Cds*F, *Cop*N), chaperons (e.g. *Ssc*1 -3, *Cds*E and *Cds*G), and a variable number of genetically diverse effectors (e.g. *tarP*, SINC), and is usually found in four clusters across the chromosome (Betts-Hampikian and Fields, 2010; Borges and Gomes, 2015; Wolff et al., 2015; Bugalhão and Mota, 2019; Hölzer et al., 2020; Heijne et al., 2021; White et al., 2021).

In *Chlamydia* spp. genomes, 5 - 8% of the CDS are estimated to be effectors (Valdivia, 2008; Bugalhão and Mota, 2019; Andersen et al., 2021). Of the T3SS effectors, TarP is an extensively studied and functionally characterised T3SS effector, responsible for remodelling the actin cytoskeleton and facilitating the entry of *Chlamydia* into the host cell (Peters et al., 2007; Bugalhão and Mota, 2019; Andersen et al., 2021). Orthologs of *tarP* are present in all *Chlamydia* spp. genomes but show extensive sequence variation. Variations in the *tarP* gene sequences are hypothesised to contribute to virulence and variations to host tissue tropisms, as shown in *C. psittaci* and *C. trachomatis*. This is thought to be due to distinct N-terminal tyrosine-repeat units and C-terminal binding domains in *C. trachomatis* TarP (Borges and Gomes, 2015; Wolff et al., 2015). Another conserved T3SS secreted protein known as SINC (secreted inner nuclear membrane–associated *Chlamydia* protein) is a functionally characterised nuclear membrane targeting protein (Mojica et al., 2015; Marschall et al., 2020), which has been identified in *C. psittaci*, *C. abortus*, *C. pecorum*, *C. caviae*, *C. felis*, *C. gallinacea*, and *C. avium* genomes (Hölzer et al., 2020). The ability of SINC to manipulate infected and non-infected host cell nuclear envelope function has been hypothesised to contribute to the high virulence of *C*. *psittaci* (Mojica et al., 2015).

#### 3.1.3 *Inc* genes

Inc proteins are virulence factors unique to the *Chlamydiae* phylum (Heinz et al., 2010). They are typified by one or more bilobed hydrophobic domains. These hydrophobic domains consist of two transmembrane helices separated by a hydrophilic loop, allowing Inc proteins to be inserted into an inclusion membrane (Dehoux et al., 2011). Inc proteins are translocated by the T3SS with the N-terminal containing the secretion signal. Inc proteins contribute to the physical structure of the inclusion membrane and perform diverse functions, including subverting vesicular trafficking for nutrient acquisition and avoiding lysosomal degradation (Elwell et al., 2016; Bugalhão and Mota, 2019).

*Inc* genes constitute a significant portion of the *Chlamydia* genome, accounting for 6% to 14% of the total coding capacity in the respective species. Comparative genomics revealed a significant variation in the number of predicted *inc* genes in different *Chlamydia* species, with 65 - 70 genes in *C. trachomatis, C. muridarum* and *C. suis*, 102 - 110 in *C. psittaci* and *C. abortus* and 140 - 147 in *C. pneumoniae* (Sigalova et al., 2019). Only a subset of Incs are conserved between species with ~23 Incs shared between *C. trachomatis*, *C. muridarum*, *C. felis*, *C. caviae* and *C. pneumoniae* (Lutter et al., 2012; Dimond et al., 2021). Of the Incs that are shared between species, sequence conservation is low among *Chlamydia* (Dehoux et al., 2011). Incs conserved across species may point to common protein functions and host interactions important in *Chlamydia* species, while non-conserved Inc proteins are hypothesised to contribute to host species differences. For instance, *Inc* genes differences account for approximately one-third of the genetic variations between *C. trachomatis* and *C. pneumoniae*. The increased number of *Inc* genes in *C. pneumoniae* may allow it to adapt to a broader host range (Dehoux et al., 2011).

In addition to species differences, Inc proteins may be responsible for differences in tissue tropisms and disease severity between *C. trachomatis* LGV and trachoma biovars. Analysis of *C. trachomatis* genomes showed that *Inc* genes were subject to positive selection with four times more non-synonymous SNPs compared to synonymous SNPs (Almeida et al., 2012; Borges and Gomes, 2015). Amino acid changes in Inc proteins were primarily specific to LGV strains and could phylogenetically separate LGV from ocular and urogenital strains (Almeida et al., 2012; Borges et al., 2012; Lutter et al., 2012). These amino acid changes occurred in regions predicted to be exposed to the host cytoplasm and thus may be involved in host-pathogen interactions and/or immune recognition. Also, LGV strains were found to have LGV-specific expression patterns from three *Inc* genes (CT058, CT192 and CT214). These expression differences correlated with specific LGV-mutations in the CT192 and CT214 promoters and within the CT059-CT058 transcript. These Inc sequence and expression differences may be associated with LGV strain tissue tropism to macrophages, possibly by inhibiting phagolysomal fusion (Almeida et al., 2012).

#### 3.1.4 *Pmp* genes

Pmps are *Chlamydiaceae* unique, membrane-bound, type V surface-exposed autotransporters, associated with many chlamydia-host cell functions, including cell adhesion and virulence. All Pmps contain N-terminal Sec-dependent leader sequences (passenger domain) with multiple short repetitive motifs (GGA(I, L, V) and FxxN), a middle region and a C-terminal autotransporter β-domain. This autotransporter β-domain is responsible for translocating the protein to the bacterial surface (Mölleken et al., 2010; Nunes et al., 2015; Vasilevsky et al., 2016). The variable chlamydial *pmp* genes are grouped into several *pmp* gene families, where some are more evolutionary conserved in sequence and gene number (such as *pmp*A, *pmp*B, *pmp*D and *pmp*H families), while others are more varied (such as *pmp*G and *pmp*E families). The subtype *pmp*G is the most expanded in gene copy numbers. Among chlamydial species, the number of *pmp* genes ranges between seven and nine genes in *C. avium*, and *C. trachomatis*, to 18 and 21 genes in *C. psittaci* and *C. pneumoniae*, accounting for approximately 14% of the coding capacity of their genomes (Vasilevsky et al., 2016; Sigalova et al., 2019; Hölzer et al., 2020). Furthermore, bioinformatic analyses have shown that the subtype *pmp*G may undergo phase variation, by exhibiting differences in the lengths of in-frame poly(G) tracts. This may lead to random, high-frequency, on/off reversible switching of gene expression, involved in host adaptation and immune evasion (Nunes et al., 2015; Sigalova et al., 2019).

#### 3.1.5 *omp*A gene

The *omp*A gene encodes for the chlamydial major outer membrane protein (MOMP), a ~40kDa surface-exposed porin protein containing four variable domains (VD I to VD IV), contributing to approximately 60% of the chlamydial outer membrane mass (Sun et al., 2007; Confer and Ayalew, 2013; Wen et al., 2016). Due to its antigenic properties, MOMP has been an attractive vaccine candidate against human and veterinary *Chlamydia* infection (Tifrea et al., 2020; Olsen et al., 2021; Quigley and Timms, 2021). The chlamydial *omp*A is a highly polymorphic gene specific to the genus *Chlamydia*, with approximately 30% of the sequence attributed to nucleotide polymorphisms and/or recombination (Nunes et al., 2009; Harris et al., 2012; Hadfield et al., 2017). Despite its highly recombinogenic nature, the chlamydial *omp*A gene is still a widely utilised marker for clinical, epidemiological, phylogenetic, and public health studies.

### 3.2 New insights into metabolic capabilities revealed by WGS

#### 3.2.1 Nucleotide scavenging and energy production genes

Comparative genomic studies commonly note differences in metabolic genes and/or lack of pathways to synthesise most amino acids, including biosynthesis of biotin, tryptophan, thiamine, folate, purines, and pyrimidines nucleotides within and among chlamydial species.

Pyrimidine and purine nucleotides are necessary for energy transduction and the biosynthesis of nucleic acids for most bacterial species. However, *Chlamydia* spp. lack the necessary genes to synthesise pyrimidine and purine nucleotides, instead hijacking host cellular systems for their energy needs (Saka and Valdivia, 2010). *Chlamydia* genomes contain species-specific differences for purine conversion genes, with only *C. caviae, C. felis, C. muridarum, C. psittaci*, and *C. pecorum* possessing a functional *guaAB-add* operon capable of producing AMP adenosine deaminase, GMP synthase and IMP dehydrogenase. Conversely, species including *C. trachomatis*, *C. gallinaceae*, *C. suis*, *C. pneumoniae*, and *C. abortus*, are lacking these conversion genes (**Figure 3**) (Voigt et al., 2012; Nunes and Gomes, 2014; Hölzer et al., 2020).

Additionally, all *Chlamydi*a genomes contain the pyrimidine interconversion genes *pyrH*, *ndk* and *pyrG* (uridylate kinase, nucleoside diphosphate kinase, and CTP synthase, respectively). These allow for the conversion of uridine monophosphate (UMP) to cytidine triphosphate (CTP). With the exception of *C. trachomatis* and *C. muridarum*, chlamydial genomes also contain the *pyr*E (orotate phosphoribosyltransferase) gene that encodes for uridine monophosphate synthetase (Saka and Valdivia, 2010; Voigt et al., 2012; Nunes and Gomes, 2014; Sigalova et al., 2019). Finally, only *C. pneumoniae* has a uridine kinase (*udk*) gene, which converts uridine or cytidine into UMP or CMP, respectively (Voigt et al., 2012; Nunes and Gomes, 2014).

*Chlamydia* utilises host-cell adenosine triphosphate (ATP), guanosine triphosphate (GTP), thymidine triphosphate (TTP) and uridine triphosphate (UTP) for survival (McClarty and Qin, 1993). Chlamydial species also contain a CTP synthetase gene (*Npt1*), which converts UTP to CTP and provides an alternative pathway for *Chlamydia* to obtain ATP (Saka and Valdivia, 2010; Sigalova et al., 2019). However, all other known mechanisms are lacking. Alternatively, WGS analyses have revealed that *Chlamydia* species contain genes for a complete glycolysis pathway (except for hexokinase, utilising hexose phosphate transporter to uptake host glucose-6-phosphate instead) and sodium-driven oxidative phosphorylation to generate its own ATP for energy or nucleic acid synthesis) and a hexose phosphate transporter, allowing *Chlamydia* to use glucose-6-phosphate as an energy source (Liang et al., 2018; Sigalova et al., 2019; Ende and Derré, 2020). However, this process would still depend on ADP availability from nucleotide uptake or interconversion.

#### 3.2.2 Tryptophan operon

Trp is an essential amino acid used by bacterial species in protein biosynthesis. This pathway has been identified in chlamydial species, and is of particular interest. Genomic analyses of *Chlamydia* spp. have identified partial/complete gene loss in the *trp* operon resulting in the selective abilities of species to synthesise Trp. In human host cells, the pro-inflammatory cytokine, interferon-gamma (IFN-γ) stimulates the indoleamine-2,3-dioxygenase (IDO) enzyme to catabolise host tryptophan to kynurenine, essentially starving the invading organism. Due to this, IFN-γ treatment can be used for infection control (Bachmann et al., 2014; Somboonna et al., 2019).

A (near) complete and functional *trp* operon, which includes *trpR-trpDCFBA* and the complementary genes *kyn*U and *prs*A, has been described in *C. caviae, C. felis*, *C. pecorum*. The *trpR* gene mediates tryptophan-dependent transcriptional repression, while the rest of the operon genes encode for anthranilate phosphoribosyltransferase (*trpD*), indole-3-glycerol phosphate synthase (*trpC*), phosphoribosylanthranilate isomerase (*trpF*), tryptophan synthase beta chain (*trpB*) and tryptophan synthase alpha chain (*trpA*). The complementary *kyn*U and *prs*A encode for kynureninase and phosphoribosylpyrophosphate. However, *C. suis* and *C. trachomatis* have only partial *trpR-trpBA* genes, while the rest of the species lack all *trp* operon genes (e.g. *C. abortus, C. gallinacea, C.* *pneumoniae* and *C. psittaci*) (Nunes et al., 2013; Bachmann et al., 2014; Hölzer et al., 2020). Interestingly, *C. pecorum* was reported to contain a *trp* operon outside the PZ, while *C. caviae* and *C. felis* have a near-full *trp* operon in the PZ. Similarly, *C. trachomatis* and *C. suis* have been reported to contain only partial *trp* operons located in their PZs (Bachmann et al., 2014; Hölzer et al., 2020). Using an example of *C. trachomatis*, where *trp* genes are organised as an operon with *trp*R separated by a 348-base pair (bp) intergenic region from *trpBA*, a recent study demonstrated that these could be independently transcribed and not necessarily expressed as a monocistronic transcript. Pokorzynski and colleagues showed that iron starvation can switch on the Trp salvage pathway, mediated by the iron-dependent repressor YtgR, which binds to an operator sequence within the *trpR-trpBA* intergenic region to repress transcription initiation from the alternative promoter for *trpBA* (Pokorzynski et al., 2019; Pokorzynski et al., 2020).

Furthermore, in ocular strains of *C. trachomatis*, the *trp* operon has a loss of function due to a single nucleotide deletion and frameshift in *trp*A gene encoding a truncated tryptophan synthase alpha (TrpA) subunit. Conversely, the *trp* operon genes of *C. trachomatis* urogenital strains (D – K) are intact and functional, having the ability to produce tryptophan from indole, further allowing them to recover from IFN-γ exposure and resume their infectious cycle and this is supported by experimental studies (Fehlner-Gardiner et al., 2002; Ziklo et al., 2016; Somboonna et al., 2019; Bommana et al., 2021). For the chlamydial species, such as *C. caviae* and *C. pecorum*, which have the complementary genes, *kyn*U and *prs*A, it has been shown that these species can synthesise tryptophan when limited in the presence of IFNγ, likely due to the presence of these genes (Wood et al., 2004; Islam et al., 2018). Overall, the biological significance of the identified *trp* polymorphisms remains uncharacterised. However, for some, one could infer with confidence, such as acquisition of *kyn*U, which mediates anthranilate synthesis from kynurenine, instead of the canonical chorismate-to-anthranilate pathway facilitated by *trp*E. The intergenic region of the *C. trachomatis trp* operon has regulatory functions that potentially optimises *trp* operon expression in specific biological contexts, including iron starvation and mucosal epithelial tropism.

#### 3.2.3 Biotin operon

Biotin is a critical cofactor involved in many central cell metabolism pathways (Fisher et al., 2012). Analysis of chlamydial genomes identified differences across species for biotin acquisition, where *C. psittaci*, *C. abortus*, *C. felis*, *C. pecorum* and *C. pneumoniae* have an intact biotin operon (*bioBFDA*) needed for *de novo* biotin synthesis. Other species, such as *C. trachomatis*, *C. caviae* and *C. muridarum* lack a functional biotin operon and thus depend on biotin salvaging (Fisher et al., 2012; Voigt et al., 2012; Nunes and Gomes, 2014; Sigalova et al., 2019). Interestingly, some species such as *C. psittaci* can synthesise biotin *de novo* and also sequester host biotin (Voigt et al., 2012).

### 3.3 Chlamydial plasmid

There is increasing evidence that the chlamydial plasmid is a key virulence factor and determinant of pathogenicity. Almost all *Chlamydia* spp., except *C. abortus* and human *C. pneumoniae* strains, contain a small (~7.5 kbp), highly conserved, non-conjugative or integrative chlamydial virulence plasmid (Pawlikowska-Warych et al., 2015; Szabo et al., 2020). Within some of the plasmid-bearing species, naturally occurring plasmid-less strains can be common, as observed in *C. pecorum* (Jelocnik et al., 2015), or rare, as seen in *C. trachomatis* (Sigar et al., 2014; Jones et al., 2020).

The chlamydial plasmids contain eight CDSs, named plasmid glycoproteins 1 to 8 (pGP 1-8), and four tandem repeat sequences, with each iteration comprising of 22 base pairs near the origin of replication (Pawlikowska-Warych et al., 2015; Zhong, 2017; Szabo et al., 2020). Briefly, CDSs 1 (pGP7) and 2 (pGP8) encode a putative integrase, involved in plasmid replication, CDS3 (pGP1) is homologous to a helicase, while CDSs 4 (pGP2) is an unknown chlamydia-specific protein. CDS5 (pGP3) encodes a secreted virulence 28 kDa protein essential for establishing persistent infection (Yang et al., 2020) and CDS 6 (pGP4) encodes a chlamydia-specific transcriptional regulator for multiple genes located within both the plasmid and chromosome including pGP3 and *glg*A, a glycogen synthase gene (Song et al., 2013). A recent study also demonstrated that in *C. trachomatis* pGP3 or pGP4 are both required for infectivity (Turman et al., 2023). Finally, CDSs7 and 8 (pGP5 and 6) are denoted as partitioning plasmid proteins (Rockey, 2011; Pawlikowska-Warych et al., 2015; Zhong, 2017; Jones et al., 2020).

Sequence and phylogenetic analyses indicate that chlamydial plasmids are evolutionarily conserved and syntenic between species, but within species, chlamydial plasmids contain unique sequence variations, further supporting the notion that chlamydial plasmids have co-evolved with the chromosome (Seth-Smith et al., 2009; Jelocnik et al., 2015; Szabo et al., 2020). In *C. trachomatis* diagnostics, the chlamydial plasmid CDSs are useful targets for diagnostic testing due to their stability, and high copy number, with up to ten copies per genome (Seth-Smith et al., 2009). However, the emergence of a urogenital mutant strain from Sweden containing a 377 bp deletion in plasmid gene CDS1 led to this plasmid locus being abolished as a diagnostic target due to false negative test results (Seth-Smith et al., 2009). This variant was characterised using WGS, and has since been occasionally reported in other countries (Piñeiro et al., 2014; Dahlberg et al., 2018; Escobedo-Guerra et al., 2019).

### 3.4 Other genomic elements

#### 3.4.1 Antibiotic resistance genes

There are limited reports of antibiotic-resistant genes (ARGs) in the genomes (including both chromosome and chlamydial plasmid) of *Chlamydia* spp. However, due to observed treatment failures, WGS studies continue to monitor for chromosomal genes and mutations associated with *Chlamydia* spp. resistance to antibiotics (Borel et al., 2016; Benamri et al., 2021).

The only ARG identified in *Chlamydia* spp. is the tetracycline resistance gene *tetA(C)* found in tetracycline-resistant *C. suis* strains, the only chlamydial species to have naturally obtained a resistance gene (Borel et al., 2016; Seth-Smith et al., 2017a). The *tetA*(C) gene encodes a tetracycline efflux pump which has been integrated as a genomic island (Tet-island) into the *C. suis* invasion-like gene (*inv*) on the chromosome. This integration most likely occurred during a transposition event directed by the transposase-encoding insertion sequence IScs605 (Joseph et al., 2016; Marti et al., 2022). The Tet-island is currently the only evidence for recent acquisition of foreign DNA from other bacterial species in *Chlamydia* as it shares high nucleotide identity with a pRAS3-type plasmid from the fish pathogen *Aeromonas salmonicida ssp. salmonicida* (Marti et al., 2022).

## 4 Recombination and horizontal gene transfer in chlamydial genomes

Due to the biphasic and intracellular life cycle of *Chlamydia* species, chlamydiae do not commonly replicate in environments occupied by other microorganisms. Most bacterial species gain foreign DNA, including antibiotic-resistance genes through environmental interactions with other bacterial species. Despite an intracellular niche, chlamydial species have developed an increased ability to undergo genomic recombination (Harris et al., 2012; Read et al., 2013). Although *Chlamydia* genomic recombination had been identified prior to 2004, Gomes et al. demonstrated that *C. trachomatis* recombination was frequent. Using phylogenetic analysis of 19 reference strains and 10 clinical isolates, two genomic regions were found to be hotspots for interstrain recombination (Gomes et al., 2007). Genetic recombination hotspots for other *Chlamydia* species have also been identified in *C. suis* (Marti et al., 2017; Seth-Smith et al., 2017a)*, C. psittaci* (Read et al., 2013; Branley et al., 2016), *C. pecorum* (White et al., 2021), and even in small regions in the otherwise monomorphic *C. abortus* (Seth-Smith et al., 2017b). Genomic regions with evidence of recombination include the genes *Inc*A, *pmp*s, *tarp* and the PZ (Gomes et al., 2004; Harris et al., 2012; Joseph et al., 2012; Borges and Gomes, 2015; Seth-Smith et al., 2021).

The most convincing evidence indicating the ability of chlamydial species to undergo genetic recombination (horizontal gene transfer (HGT)) was reported in co-culture studies (Marti et al., 2022). Co-culture of *C. suis* carrying the *tetC* gene with *C. trachomatis*, *C. muridarum*, and *C. caviae* confirmed genomic integration of the *tetC* island in both *C. trachomatis* and *C. muridarum* (but not *C. caviae*) (Suchland et al., 2009). Similarly, chlamydial interspecies HGT was analysed using crosses of tetracycline (Tc)-resistant *C. trachomatis* L2/434 and chloramphenicol (Cam)-resistant *C. muridarum* VR-123. WGS of the recombinant clones identified examples of duplications, mosaic recombination endpoints, and recombined sequences that were not linked to the selection marker (Suchland et al., 2019). A more recent study co-cultured tetracycline-resistant *C. suis* with rifamycin group-resistant *C. suis* resulting in an overall *in vitro* recombination efficiency of 28% (Marti et al., 2021). However, these observations have not been found in clinical isolates, and tetracyclines still remain part of the first-line defence antibiotics for human chlamydial infections.

## 5 Whole genome phylogenies resolve fine-detail relationships between *Chlamydia* species and strains

**Figure 4:**
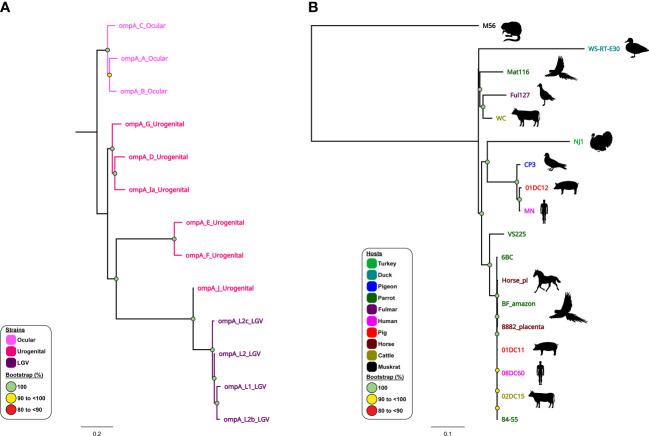
Phylogenomic relationships of *C trachomatis* and *C psittaci* reference strains. Midpoint-rooted maximum-likelihood (ML) core-genome phylogenetic trees constructed using Parsnp version 1.2 of **(A)** 13 aligned publicly available complete *C trachomatis* genomes; and **(B)** 18 aligned publicly available complete *C psittaci* genomes. For *C trachomatis*, genome sequences are coloured according to tissue tropism/known genotypes (as depicted in the figure legend). For *C psittaci*, genome sequences are coloured according to their respective host (as depicted in the figure legend). Corresponding host silhouettes are shown adjacent to sequence names. Bootstrap values greater than 0.8 are shown. Branch lengths represent the nucleotide substitutions per site, as indicated by the scale bar.

The use of WGS combined with sample-specific metadata have provided important observations on the contemporary history of current *C. trachomatis* circulating lineages within global populations (Andersson et al., 2016; Hadfield et al., 2017; Seth-Smith et al., 2021). It is well established that *C. trachomatis* is comprised of three distinct lineages, where *omp*A genotypes A, B, Ba and C are associated with trachoma, genotypes D-K associated with non-invasive UGT infections, and genotypes L1-L3 and L2b associated with invasive infections and LGV (Harris et al., 2012; Joseph et al., 2012; Andersson et al., 2016; Hadfield et al., 2017) (**Figure 4A**). However, phylogenomic studies show that not all strains may fit into this traditional separation, perhaps reflecting *C. trachomatis* adaptation to their respective niche and the noted recombination events. For example, whole-genome phylogenetic analyses of ocular isolates obtained from Australian Aboriginal people with trachoma placed these isolates into two lineages that fall outside the classical trachoma lineage (A-C). Instead, these genetically distinct strains clustered within lineages that were previously occupied exclusively by UGT isolates (namely genotypes D–K). These trachoma isolates appear to be recombinants with the *C. trachomatis* UGT genome backbones, where recombination was noted in *omp*A and *pmp* loci replacing them with those *omp*A and *pmp* genotypes characteristic of ocular isolates (Andersson et al., 2016). Similarly, recent evidence has suggested that clinically relevant recombination events have occurred between two *C. trachomatis* strains (serovar D and LGV). Recent outbreaks of LGV (L2b strain) within men who have sex with men (MSM) populations have resulted in genetic recombination of the *omp*A gene from non-LGV strains (Borges et al., 2019; Borges et al., 2021). A previously undetected outbreak of LGV may have gone undiagnosed due to over- reliance on the *omp*A gene for identification. Further genomic analysis found that the *C. trachomatis* L2B strain had undergone genetic recombination, acquiring *omp*A from the non-LGV strain *C. trachomatis* D-Da (Borges et al., 2019). Furthermore, follow-up studies have indicated that this L2b/D-Da variant is spreading across Europe and, with the continued reliance on *omp*A for strain identification, the probability of further dissemination across the globe is high (Borges et al., 2021).

Similarly, phylogenomic analyses confirmed that modern human *C. pneumoniae* strains have evolved separately from animal strains (Roulis et al., 2015b), while further demonstrating a distinct Australian indigenous *C. pneumoniae* clade pre-dating European exploration of the continent (Roulis et al., 2015a). Phylogenomic analyses have demonstrated that whilst the global *C. psittaci* population is genetically diverse, there are also recently emerged, globally distributed and highly clonal lineages (such as *C. psittaci* ST24 strains) that infect humans, parrots, and, most recently, horses. Within this highly clonal ST24 lineage, the equine, human and parrot strains differ by less than 200 single nucleotide variants (SNVs), evenly distributed around the highly conserved and syntenic chromosome (Read et al., 2013; Branley et al., 2016; Jenkins et al., 2018; White et al., 2022; White et al., 2023). However, between genetically diverse *C. psittaci* lineages (such as ST24 and pigeon-associated clade) SNVs and differences in gene content were noted in distinct chromosomal regions, including T3SS, *omp*A, *Inc* and *pmp* genes (Voigt et al., 2012; Wolff et al., 2015; Hölzer et al., 2020; Vorimore et al., 2021a) (**Figure 4B**). The difference in gene content and SNVs likely plays a role in *C. psittaci* host tropism, adaptation and species-specific pathogenicity (Wolff et al., 2015; Favaroni et al., 2021). Furthermore, other phylogenomic analyses have genetically separated distinct avian-type *C. abortus* strains from the traditional ruminant *C. abortus* and/or avian *C. psittaci* strains (Longbottom et al., 2021; Zaręba-Marchewka et al., 2021) and confirmed that ruminant *C. abortus* genomic diversity is low level with no recombination detected, contrasting other related species (Seth-Smith et al., 2017b).

### 5.1 Complementing WGS studies: Gene-centric molecular epidemiology of chlamydial infections

WGS is considered the norm for evolutionary and epidemiological studies of many pathogens, including the human pathogen *C. trachomatis*, with the most available WGS data among chlamydial genomes (Harris et al., 2012; Bowden et al., 2021; Seth-Smith et al., 2021). However, numerous molecular epidemiology and/or genetic diversity studies of *C. trachomatis* and veterinary species still rely on gene-centric typing methods using a single (e.g. *omp*A) or multiple conserved gene markers (e.g. multi locus sequence typing (MLST)) due to decreased costs. Genotyping using the full-length as well as fragments of the highly variable *omp*A gene is employed in genetic diversity studies for veterinary and human chlamydial infections, as it provides an initial molecular characterisation of the infecting strains (Liu et al., 2019; Rawre et al., 2019; Robbins et al., 2019).

**Figure 5:**
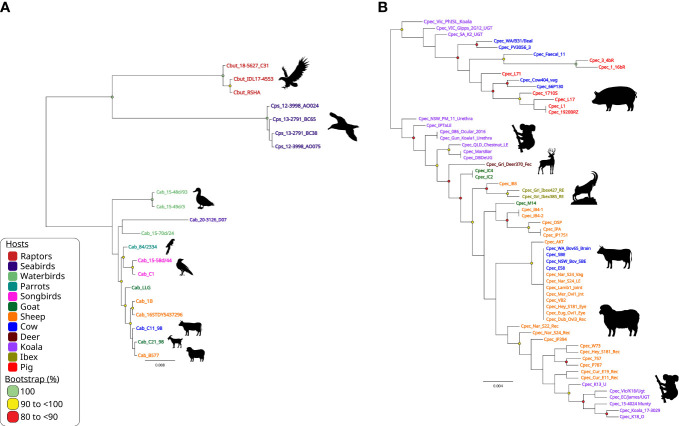
*Chlamydia* intraspecies genetic diversity. Midpoint-rooted maximum-likelihood (ML) phylogenetic analysis of 3,098 bp concatenated MLST sequences alignment representing **(A)** 20 avian and livestock STs from closely related *C buteonis*, *C psittaci*, traditional livestock and novel avian *C abortus*; and **(B)** 62 C*. pecorum* STs from a range of hosts. Phylogenetic trees were constructed using FastTree version 2.1.11, as implemented in Geneious Prime (available at: https://www.geneious.com/). Corresponding host silhouettes are shown adjacent to sequence names. Bootstrap values greater than 0.8 are shown. Branch lengths represent the nucleotide substitutions per site, as indicated by the scale bar.

However, the insufficient resolution and noted recombination in the chlamydial *omp*A genes, led to use of species-specific higher-resolution genotyping methods, such as MLST (Versteeg et al., 2018a; Jelocnik et al., 2019). The chlamydial MLST is globally adopted as a rapid, universal fine-detailed molecular typing tool for both human *C. trachomatis* and veterinary chlamydial pathogens (**Figure 5**). As a technically easier and cheaper alternative to WGS, MLST utilises the Chlamydiales PubMLST database (http://pubMLST.org/chlamydiales) hosted on a platform that provides easy to use sequence and phylogenetic analyses. Furthermore, WGS studies are often supplemented with extended genotyping of samples using MLST due to high congruency with WGS phylogenetic clustering (Guo et al., 2017; Jenkins et al., 2018). Core genome and/or traditional MLST-derived phylogeny is highly congruent with WGS and/or SNP-derived phylogenies, and avoids the need to reconstruct computationally heavy phylogenies (Jolley et al., 2018; Patiño et al., 2018; Versteeg et al., 2018a; Jelocnik et al., 2019; Floridia-Yapur et al., 2021). Chlamydial MLST was effectively used in uncovering the global epidemiology of *C. trachomatis* strains (Herrmann et al., 2015; Danielewski et al., 2017), identifying clonal psittacine, horse and human strains of *C. psittaci* during an outbreak (Anstey et al., 2021) as well closely related but genetically diverse avian chlamydial strains (**Figure 5A**), distinguishing diverse koala *C. pecorum* strains to aid in translocation of animals (**Figure 5B**) (Fernandez et al., 2019) and other examples (Batteiger et al., 2014).

## 6 Complementing WGS studies: Multi-omics integration for a systems biology understanding of chlamydial infections

**Figure 6:**
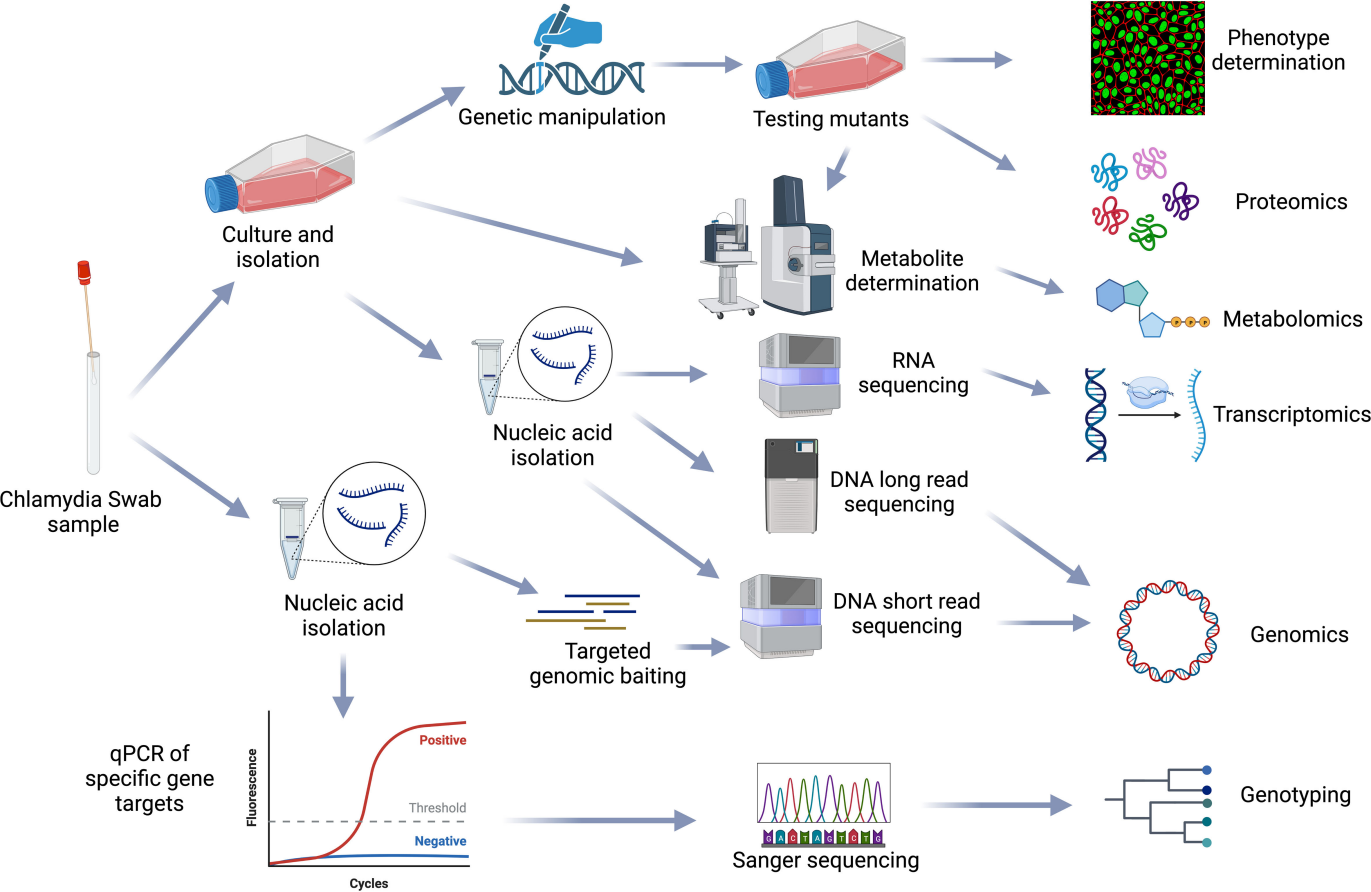
Chlamydial omics. Representation of the workflows from sample processing to multiple omics analysis methods used in the chlamydial field. Figure created using BioRender (available from https://www.biorender.com/).

Systems biology is the large-scale study of how genes, proteins, metabolites and other regulatory elements in an organism interact together using various integrated high-throughput multi-omic techniques, including genomics, transcriptomics, proteomics and metabolomics. Due to their biphasic, intracellular lifecycle, *Chlamydia* remain difficult to genetically manipulate; multi-omic approaches to understanding *Chlamydia* biology and pathogenesis are thus essential (**Figure 6**).

Genomics forms the basis for systems biology and allows genes/proteins expressed during different conditions to be mapped and identified using complementary transcriptomic and proteomic methods. In *Chlamydia*, these approaches have provided insights into homologous virulence factor expression differences between *C. psittaci* and *C. abortus* which may be related to disease severity in humans (Beder and Saluz, 2018). Transcriptomics/proteomics studies have also revealed the host and pathogen genes expressed during the three phases of chlamydia infection- early, mid and late (Albrecht et al., 2010; Humphrys et al., 2013; Hayward et al., 2019), the chlamydial gene expression changes in response to stress such as iron limitation and exposure to IFN-γ (Belland et al., 2003; Brinkworth et al., 2018) and how *C. trachomatis* alters host chromatin accessibility by inducing epigenetic modifications (Hayward et al., 2020). These studies also led to the identification of new sRNAs in *C. trachomatis* (Albrecht et al., 2010), the role of the chlamydial plasmid in infection (Porcella et al., 2015) and insights into chlamydial-induced epithelial to mesenchymal cell transition (Zadora et al., 2019). It has also provided key insights into expression differences between EBs and RBs in *C. trachomatis* including increased T3SS proteins in EBs (Østergaard et al., 2016; Skipp et al., 2016). Recently, genomics was used to reconstruct the first *Chlamydia* genome-scale metabolic model (GSMM). GSMMs are mathematical models used to computationally describe the movement of metabolites within an organism. This model was then integrated with proteomic data to elucidate metabolic differences and altered flux between EBs and RBs (Yang et al., 2019).

Finally, multi-omics methods have also been essential for vaccine development and the discovery of new drug targets against *Chlamydia*. Immunoproteomics have been used to identify new *C. trachomatis* T-cell antigens for vaccine development and to identify immunogenic proteins in *C. abortus* outer membrane complexes (Karunakaran et al., 2008; Longbottom et al., 2019). The use of RNA-seq and metabolomics identified essential host metabolites and pathways for chlamydia infection, such as glutamine and guanine nucleotide biosynthesis. Targeting of these host-pathways was shown to inhibit chlamydia infection (Rother et al., 2018; Rajeeve et al., 2020).

Currently, most multi-omics approaches in *Chlamydia* involve characterising gene expression at the bulk population level from thousands to millions of *Chlamydia* and/or host cells within a given sample (Hayward et al., 2021; Pokorzynski et al., 2022). However, this approach ignores the important variations between individual cells. Future single-cell and spatial approaches such as single-cell RNA-sequencing (scRNA-seq) in bacteria (Kuchina et al., 2021) and/or host cells (Hayward et al., 2019) for interrogating host-pathogen responses to chlamydia infections may reveal meaningful differences in cell-to-cell responses to and/or from *Chlamydia* as well as identify the spatial distribution of important cell populations/lineages during infection. These existing and future omics techniques can be applied to *Chlamydia* infected tissues or advanced organotypic/organoid models, which better represent the host factors encountered (Nogueira et al., 2017; Versteeg et al., 2018b; McQueen et al., 2020; Dolat and Valdivia, 2021; Dolat et al., 2022; Edwards et al., 2022). This may provide insight into the pathogenesis *in vivo* and shed light on differences in genes or virulence factors required when infecting different host cells.

## 7 Future directions

The members of family *Chlamydiaceae* are an important species in the list of intracellular bacteria. They are highly adapted to their environments and depend on host cell machinery to survive and replicate. Their host range is one of the largest known, with predictions of a chlamydial species for every cell type on the planet (Collingro et al., 2020). With respect to *C. trachomatis*, it is the most common sexually transmitted infection and results in millions of cases of infertility and blindness across the globe. Improvements in WGS techniques have given rise to a deeper understanding of the chlamydial genome and the specific adaptations that have allowed specific species to thrive in different host environments. WGS has also resulted in new understandings of genetic recombination within species (but also interspecies) and has highlighted the issues around single gene sequencing to predict species evolution. In addition to enriching our understanding of *Chlamydia*, advances in genomics provides opportunities for rapid diagnosis of chlamydial infections and antimicrobial resistance detection using targeted single-gene nanopore sequencing or direct metagenome sequencing (Gu et al., 2020; Zhou et al., 2022). The advantages of using genomics for diagnosis is the ability to reveal the presence or absence of different virulence and antimicrobial-resistant genes, plasmids or important lineage/phylogenetic markers that may guide treatment and/or outbreak management.

The increasing number of chlamydial genomes sequenced and in publicly available databases poses challenges for future genome analysis as phylogenetic trees become more computationally intensive to construct. Thus, the future development of a *Chlamydia* hierarchical genome typing scheme based on core genome MLST or SNPs, such as those developed for *C. trachomatis* (Patiño et al., 2018; Versteeg et al., 2018a) will provide a standardised, universal and stable method for rapid chlamydia genotyping and disease surveillance. These hierarchical schemes can rapidly assign sequence types to infer phylogenetic relationships, require less intensive computation, and are made up of multiple SNPs or MLST schemes with increasing resolution to provide both short and long-term epidemiological information for outbreak detection and long-term lineage tracking, respectively. Many aspects of the *Chlamydiae* are still hidden, but with more and more genome-scale data published every day, our understanding of this complex and widespread bacterial species continues to improve.

## Author contributions

Conceptualisation: MJ, LL, GM. Formal analysis: LL, SP, VK, GM and MJ. Data analysis: LL, SP, VK and MJ. Writing, original draft preparation: LL and MJ. Writing, figure preparation,: LL, VK and SP. Writing, review and editing: LL, SP, VK, GM and MJ. All authors have contributed to the article and approved the submitted version.

## References

[B1] Albrecht M. Sharma C. M. Reinhardt R. Vogel J. Rudel T. (2010). Deep sequencing-based discovery of the *Chlamydia trachomatis* transcriptome. Nucleic Acids Res. 38 (3), 868–877. doi: 10.1093/nar/gkp1032 19923228 PMC2817459

[B2] Almeida F. Borges V. Ferreira R. Borrego M. J. Gomes J. P. Mota L. J. (2012). Polymorphisms in inc proteins and differential expression of *inc* genes among *Chlamydia trachomatis* strains correlate with invasiveness and tropism of lymphogranuloma venereum isolates. J. Bacteriol. 194 (23), 6574–6585. doi: 10.1128/jb.01428-12 23042990 PMC3497493

[B3] Andersen S. E. Bulman L. M. Steiert B. Faris R. Weber M. M. (2021). Got mutants? how advances in chlamydial genetics have furthered the study of effector proteins. Pathog. Dis. 79 (2), ftaa078. doi: 10.1093/femspd/ftaa078 33512479 PMC7862739

[B4] Andersson P. Harris S. R. Smith H. M. B. S. Hadfield J. O'Neill C. Cutcliffe L. T.. (2016). *Chlamydia trachomatis* from Australian aboriginal people with trachoma are polyphyletic composed of multiple distinctive lineages. Nat. Commun. 7, 10688–10688. doi: 10.1038/ncomms10688 26912299 PMC4773424

[B5] Anstey S. I. Kasimov V. Jenkins C. Legione A. Devlin J. Amery-Gale J.. (2021). *Chlamydia psittaci* ST24: clonal strains of one health importance dominate in Australian horse, bird and human infections. Pathogens 10 (8), 1015. doi: 10.3390/pathogens10081015 34451478 PMC8401489

[B6] Bachmann N. L. Polkinghorne A. Timms P. (2014). *Chlamydia* genomics: providing novel insights into chlamydial biology. Trends Microbiol. 22 (8), 464–472. doi: 10.1016/j.tim.2014.04.013 24882432

[B7] Bachmann N. L. Sullivan M. J. Jelocnik M. Myers G. S. Timms P. Polkinghorne A. (2015). Culture-independent genome sequencing of clinical samples reveals an unexpected heterogeneity of infections by *Chlamydia pecorum* . J. Clin. Microbiol. 53 (5), 1573–1581. doi: 10.1128/jcm.03534-14 25740768 PMC4400774

[B8] Bastidas R. J. Valdivia R. H. (2016). Emancipating *Chlamydia*: advances in the genetic manipulation of a recalcitrant intracellular pathogen. Microbiol. Mol. Biol. Rev. 80 (2), 411–427. doi: 10.1128/MMBR.00071-15 27030552 PMC4867370

[B9] Batteiger B. E. Wan R. Williams J. A. He L. Ma A. Fortenberry J. D.. (2014). Novel *Chlamydia trachomatis* strains in heterosexual sex partners, Indianapolis, Indiana, USA. Emerg. Infect. Dis. 20 (11), 1841–1847. doi: 10.3201/2011.140604 25340463 PMC4214310

[B10] Beder T. Saluz H. P. (2018). Virulence-related comparative transcriptomics of infectious and non-infectious chlamydial particles. BMC Genom. 19 (1), 575. doi: 10.1186/s12864-018-4961-x PMC6090853 30068313

[B11] Belland R. J. Nelson D. E. Virok D. Crane D. D. Hogan D. Sturdevant D.. (2003). Transcriptome analysis of chlamydial growth during IFN-gamma-mediated persistence and reactivation. Proc. Natl. Acad. Sci. U.S.A. 100 (26), 15971–15976. doi: 10.1073/pnas.2535394100 14673075 PMC307677

[B12] Benamri I. Azzouzi M. Sanak K. Moussa A. Radouani F. (2021). An overview of genes and mutations associated with chlamydiae species' resistance to antibiotics. Ann. Clin. Microbiol. Antimicrob. 20 (1), 59. doi: 10.1186/s12941-021-00465-4 34479551 PMC8414684

[B13] Betts-Hampikian H. Fields K. (2010). The chlamydial type III secretion mechanism: revealing cracks in a tough nut. Front. Microbiol. 1. doi: 10.3389/fmicb.2010.00114 PMC3125583 21738522

[B14] Bommana S. Somboonna N. Richards G. Tarazkar M. Dean D. (2021). Tryptophan operon diversity reveals evolutionary trends among geographically disparate *Chlamydia trachomatis* ocular and urogenital strains affecting tryptophan repressor and synthase function. mBio 12 (3), e00605–e00621. doi: 10.1128/mBio.00605-21 33975934 PMC8262981

[B15] Borel N. Leonard C. Slade J. Schoborg R. V. (2016). Chlamydial antibiotic resistance and treatment failure in veterinary and human medicine. Curr. Clin. Microbiol. Rep. 3, 10–18. doi: 10.1007/s40588-016-0028-4 27218014 PMC4845085

[B16] Borges V. Cordeiro D. Salas A. I. Lodhia Z. Correia C. Isidro J.. (2019). *Chlamydia trachomatis*: when the virulence-associated genome backbone imports a prevalence-associated major antigen signature. Microb. Genom. 5 (11), e000313. doi: 10.1099/mgen.0.000313 31697227 PMC6927300

[B17] Borges V. Gomes J. P. (2015). Deep comparative genomics among *Chlamydia trachomatis* lymphogranuloma venereum isolates highlights genes potentially involved in pathoadaptation. Infect. Genet. Evol. 32, 74–88. doi: 10.1016/j.meegid.2015.02.026 25745888

[B18] Borges V. Nunes A. Ferreira R. Borrego M. J. Gomes J. P. (2012). Directional evolution of *Chlamydia trachomatis* towards niche-specific adaptation. J. Bacteriol. 194 (22), 6143–6153. doi: 10.1128/jb.01291-12 22961851 PMC3486361

[B1001] Borges V. Isidro J. Correia C. Cordeiro D. Vieira L. Lodhia Z. (2021). Transcontinental dissemination of the L2b/D-Da recombinant chlamydia trachomatis lymphogranuloma venereum (LGV) strain: Need of broad multi-country molecular surveillance. Clin. Infect. Dis. 73 (4), e1004–e1007. doi: 10.1093/cid/ciab067 33512482

[B19] Bowden K. E. Joseph S. J. Cartee J. C. Ziklo N. Danavall D. Raphael B. H.. (2021). Whole-genome enrichment and sequencing of *Chlamydia trachomatis* directly from patient clinical vaginal and rectal swabs. mSphere 6 (2), e01302–e01320. doi: 10.1128/mSphere.01302-20 33658279 PMC8546720

[B20] Branley J. Bachmann N. L. Jelocnik M. Myers G. S. A. Polkinghorne A. (2016). Australian Human and parrot *Chlamydia psittaci* strains cluster within the highly virulent 6BC clade of this important zoonotic pathogen. Sci. Rep. 6, 30019–30019. doi: 10.1038/srep30019 27488134 PMC4973220

[B21] Brinkworth A. J. Wildung M. R. Carabeo R. A. (2018). Genomewide transcriptional responses of iron-starved *Chlamydia trachomatis* reveal prioritization of metabolic precursor synthesis over protein translation. mSystems 3 (1), e00184–17. doi: 10.1128/mSystems.00184-17 29468197 PMC5811630

[B22] Bugalhão J. N. Mota L. J. (2019). The multiple functions of the numerous *Chlamydia trachomatis* secreted proteins: the tip of the iceberg. Microb. Cell 6 (9), 414–449. doi: 10.15698/mic2019.09.691 31528632 PMC6717882

[B23] Carlson J. H. Hughes S. Hogan D. Cieplak G. Sturdevant D. E. McClarty G.. (2004). Polymorphisms in the *Chlamydia trachomatis* cytotoxin locus associated with ocular and genital isolates. Infect. Immun. 72 (12), 7063–7072. doi: 10.1128/iai.72.12.7063-7072.2004 15557630 PMC529170

[B24] Cheong H. C. Lee C. Y. Q. Cheok Y. Y. Tan G. M. Y. Looi C. Y. Wong W. F. (2019). *Chlamydiaceae*: diseases in primary hosts and zoonosis. Microorganisms 7 (5), 146. doi: 10.3390/microorganisms7050146 31137741 PMC6560403

[B25] Chiarelli T. J. Grieshaber N. A. Omsland A. Remien C. H. Grieshaber S. S. (2020). Single-inclusion kinetics of *Chlamydia trachomatis* development. mSystems 5 (5), e00689–20. doi: 10.1128/mSystems.00689-20 33051378 PMC7567582

[B26] Christiansen M. T. Brown A. C. Kundu S. Tutill H. J. Williams R. Brown J. R.. (2014). Whole-genome enrichment and sequencing of *Chlamydia trachomatis* directly from clinical samples. BMC Infect. Dis. 14, 591. doi: 10.1186/s12879-014-0591-3 25388670 PMC4233057

[B27] Collingro A. Köstlbacher S. Horn M. (2020). *Chlamydiae* in the environment. Trends Microbiol. 28 (11), 877–888. doi: 10.1016/j.tim.2020.05.020 32591108

[B28] Collingro A. Tischler P. Weinmaier T. Penz T. Heinz E. Brunham R. C.. (2011). Unity in variety–the pan-genome of the *Chlamydiae* . Mol. Biol. Evol. 28 (12), 3253–3270. doi: 10.1093/molbev/msr161 21690563 PMC3247790

[B29] Confer A. W. Ayalew S. (2013). The OmpA family of proteins: roles in bacterial pathogenesis and immunity. Vet. Microbiol. 163 (3-4), 207–222. doi: 10.1016/j.vetmic.2012.08.019 22986056

[B30] da Cunha M. Pais S. V. Bugalhão J. N. Mota L. J. (2017). The *Chlamydia trachomatis* type III secretion substrates CT142, CT143, and CT144 are secreted into the lumen of the inclusion. PloS One 12 (6), e0178856. doi: 10.1371/journal.pone.0178856 28622339 PMC5473537

[B31] Dahlberg J. Hadad R. Elfving K. Larsson I. Isaksson J. Magnuson A.. (2018). Ten years transmission of the new variant of *Chlamydia trachomatis* in Sweden: prevalence of infections and associated complications. Sex. Transm. Infect. 94 (2), 100–104. doi: 10.1136/sextrans-2016-052992 28724744 PMC5870454

[B32] Danielewski J. A. Phillips S. Kong F. Y. S. Smith K. S. Hocking J. S. Guy R.. (2017). A snapshot of *Chlamydia trachomatis* genetic diversity using multilocus sequence type analysis in an Australian metropolitan setting. Eur. J. Clin. Microbiol. Infect. Dis. 36 (7), 1297–1303. doi: 10.1007/s10096-017-2935-6 28220321

[B33] Dehoux P. Flores R. Dauga C. Zhong G. Subtil A. (2011). Multi-genome identification and characterization of *Chlamydiae*-specific type III secretion substrates: the inc proteins. BMC Genom. 12, 109. doi: 10.1186/1471-2164-12-109 PMC3048545 21324157

[B34] Dharamshi J. E. Köstlbacher S. Schön M. E. Collingro A. Ettema T. J. G. Horn M. (2023). Gene gain facilitated endosymbiotic evolution of *Chlamydiae* . Nat. Microbiol. 8 (1), 40–54. doi: 10.1038/s41564-022-01284-9 36604515 PMC9816063

[B35] Dharamshi J. E. Tamarit D. Eme L. Stairs C. W. Martijn J. Homa F.. (2020). Marine sediments illuminate *Chlamydiae* diversity and evolution. Curr. Biol. 30 (6), 1032–1048.e1037. doi: 10.1016/j.cub.2020.02.016 32142706

[B36] Dimond Z. E. Suchland R. J. Baid S. LaBrie S. D. Soules K. R. Stanley J.. (2021). Inter-species lateral gene transfer focused on the *Chlamydia* plasticity zone identifies loci associated with immediate cytotoxicity and inclusion stability. Mol. Microbiol. 116 (6), 1433–1448. doi: 10.1111/mmi.14832 34738268 PMC9119408

[B37] Dolat L. Carpenter V. K. Chen Y. S. Suzuki M. Smith E. P. Kuddar O.. (2022). *Chlamydia* repurposes the actin-binding protein EPS8 to disassemble epithelial tight junctions and promote infection. Cell Host Microbe 30 (12), 1685–1700.e1610. doi: 10.1016/j.chom.2022.10.013 36395759 PMC9793342

[B38] Dolat L. Valdivia R. H. (2021). An endometrial organoid model of interactions between *Chlamydia* and epithelial and immune cells. J. Cell Sci. 134 (5), 1685–1700. doi: 10.1242/jcs.252403 PMC7970307 33468625

[B39] Edwards V. L. McComb E. Gleghorn J. P. Forney L. Bavoil P. M. Ravel J. (2022). Three-dimensional models of the cervicovaginal epithelia to study host-microbiome interactions and sexually transmitted infections. Pathog. Dis. 80 (1), ftac026. doi: 10.1093/femspd/ftac026 35927516 PMC9419571

[B40] Edwards V. L. Smith S. B. McComb E. J. Tamarelle J. Ma B. Humphrys M. S.. (2019). The cervicovaginal microbiota-host interaction modulates *Chlamydia trachomatis* infection. mBio 10 (4), e01548–19. doi: 10.1128/mBio.01548-19 31409678 PMC6692509

[B41] Elwell C. Mirrashidi K. Engel J. (2016). *Chlamydia* cell biology and pathogenesis. Nat. Rev. Microbiol. 14 (6), 385–400. doi: 10.1038/nrmicro.2016.30 27108705 PMC4886739

[B42] Ende R. J. Derré I. (2020). Host and bacterial glycolysis during *Chlamydia trachomatis* infection. Infect. Immun. 88 (12), e00545–e00520. doi: 10.1128/IAI.00545-20 32900818 PMC7671904

[B43] Escobedo-Guerra M. R. Katoku-Herrera M. Lopez-Hurtado M. Villagrana-Zesati J. R. de Haro-Cruz M. J. Guerra-Infante F. M. (2019). Identification of a new variant of *Chlamydia trachomatis* in Mexico. Enferm Infecc Microbiol. Clin. 37 (2), 93–99. doi: 10.1016/j.eimc.2018.02.008 29636285

[B44] Favaroni A. Trinks A. Weber M. Hegemann J. H. Schnee C. (2021). Pmp repertoires influence the different infectious potential of avian and mammalian *Chlamydia psittaci* strains. Front. Microbiol. 12. doi: 10.3389/fmicb.2021.656209 PMC8039305 33854490

[B45] Fehlner-Gardiner C. Roshick C. Carlson J. H. Hughes S. Belland R. J. Caldwell H. D.. (2002). Molecular basis defining human *Chlamydia trachomatis* tissue tropism. a possible role for tryptophan synthase. J. Biol. Chem. 277 (30), 26893–26903. doi: 10.1074/jbc.M203937200 12011099

[B46] Fernandez C. M. Schmertmann L. J. Higgins D. P. Casteriano A. Irinyi L. Mella V. S. A.. (2019). Genetic differences in *Chlamydia pecorum* between neighbouring sub-populations of koalas (*Phascolarctos cinereus*). Vet. Microbiol. 231, 264–270. doi: 10.1016/j.vetmic.2019.02.020 30853132

[B47] Fisher D. J. Fernández R. E. Adams N. E. Maurelli A. T. (2012). Uptake of biotin by *Chlamydia* spp. through the use of a bacterial transporter (BioY) and a host-cell transporter (SMVT). PloS One 7 (9), e46052. doi: 10.1371/journal.pone.0046052 23029384 PMC3459881

[B48] Floridia-Yapur N. Rusman F. Diosque P. Tomasini N. (2021). Genome data vs MLST for exploring intraspecific evolutionary history in bacteria: much is not always better. Infect. Genet. Evol. 93, 104990. doi: 10.1016/j.meegid.2021.104990 34224899

[B49] Gitsels A. Sanders N. Vanrompay D. (2019). Chlamydial infection from outside to inside. Front. Microbiol. 10. doi: 10.3389/fmicb.2019.02329 PMC6795091 31649655

[B50] Gomes J. P. Bruno W. J. Borrego M. J. Dean D. (2004). Recombination in the genome of *Chlamydia trachomatis* involving the polymorphic membrane protein c gene relative to *omp*A and evidence for horizontal gene transfer. J. Bacteriol 186 (13), 4295–4306. doi: 10.1128/jb.186.13.4295-4306.2004 15205432 PMC421610

[B51] Gomes J. P. Bruno W. J. Nunes A. Santos N. Florindo C. Borrego M. J.. (2007). Evolution of *Chlamydia trachomatis* diversity occurs by widespread interstrain recombination involving hotspots. Genome Res. 17 (1), 50–60. doi: 10.1101/gr.5674706 17090662 PMC1716266

[B52] Gu L. Liu W. Ru M. Lin J. Yu G. Ye J.. (2020). The application of metagenomic next-generation sequencing in diagnosing *Chlamydia psittaci* pneumonia: a report of five cases. BMC Pulm. Med. 20 (1), 65. doi: 10.1186/s12890-020-1098-x 32178660 PMC7077129

[B53] Guo W. Jelocnik M. Li J. Sachse K. Polkinghorne A. Pannekoek Y.. (2017). From genomes to genotypes: molecular epidemiological analysis of *Chlamydia gallinacea* reveals a high level of genetic diversity for this newly emerging chlamydial pathogen. BMC Genom. 18 (1), 949. doi: 10.1186/s12864-017-4343-9 PMC5717833 29212448

[B54] Hadfield J. Harris S. R. Seth-Smith H. M. B. Parmar S. Andersson P. Giffard P. M.. (2017). Comprehensive global genome dynamics of *Chlamydia trachomatis* show ancient diversification followed by contemporary mixing and recent lineage expansion. Genome Res. 27 (7), 1220–1229. doi: 10.1101/gr.212647.116 28588068 PMC5495073

[B55] Harris S. R. Clarke I. N. Seth-Smith H. M. B. Solomon A. W. Cutcliffe L. T. Marsh P.. (2012). Whole-genome analysis of diverse *Chlamydia trachomatis* strains identifies phylogenetic relationships masked by current clinical typing. Nat. Genet. 44 (4), 413–419. doi: 10.1038/ng.2214 22406642 PMC3378690

[B56] Hayward R. J. Humphrys M. S. Huston W. M. Myers G. S. A. (2021). Dual RNA-seq analysis of *in vitro* infection multiplicity and RNA depletion methods in *Chlamydia*-infected epithelial cells. Sci. Rep. 11 (1), 10399. doi: 10.1038/s41598-021-89921-x 34001998 PMC8128910

[B57] Hayward R. J. Marsh J. W. Humphrys M. S. Huston W. M. Myers G. S. A. (2019). Early transcriptional landscapes of *Chlamydia trachomatis*-infected epithelial cells at single cell resolution. Front. Cell Infect. Microbiol. 9. doi: 10.3389/fcimb.2019.00392 PMC6877545 31803632

[B58] Hayward R. J. Marsh J. W. Humphrys M. S. Huston W. M. Myers G. S. A. (2020). Chromatin accessibility dynamics of *Chlamydia*-infected epithelial cells. Epigenet. Chromatin 13 (1), 45. doi: 10.1186/s13072-020-00368-2 PMC7590614 33109274

[B59] Heijne M. Jelocnik M. Umanets A. Brouwer M. S. M. Dinkla A. Harders F.. (2021). Genetic and phenotypic analysis of the pathogenic potential of two novel *Chlamydia gallinacea* strains compared to *Chlamydia psittaci* . Sci. Rep. 11 (1), 16516. doi: 10.1038/s41598-021-95966-9 34389764 PMC8363750

[B60] Heinz E. Rockey D. D. Montanaro J. Aistleitner K. Wagner M. Horn M. (2010). Inclusion membrane proteins of *Protochlamydia amoebophila* UWE25 reveal a conserved mechanism for host cell interaction among the *Chlamydiae* . J. Bacteriol. 192 (19), 5093–5102. doi: 10.1128/jb.00605-10 20675479 PMC2944539

[B61] Herrmann B. Isaksson J. Ryberg M. Tångrot J. Saleh I. Versteeg B.. (2015). Global multilocus sequence type analysis of *Chlamydia trachomatis* strains from 16 countries. J. Clin. Microbiol. 53 (7), 2172–2179. doi: 10.1128/JCM.00249-15 25926497 PMC4473235

[B62] Hölzer M. Barf L.-M. Lamkiewicz K. Vorimore F. Lataretu M. Favaroni A.. (2020). Comparative genome analysis of 33 *Chlamydia* strains reveals characteristic features of *Chlamydia psittaci* and closely related species. Pathogens 9 (11), 899. doi: 10.3390/pathogens9110899 33126635 PMC7694038

[B63] Humphrys M. S. Creasy T. Sun Y. Shetty A. C. Chibucos M. C. Drabek E. F.. (2013). Simultaneous transcriptional profiling of bacteria and their host cells. PloS One 8 (12), e80597. doi: 10.1371/journal.pone.0080597 24324615 PMC3851178

[B64] Islam M. M. Jelocnik M. Huston W. M. Timms P. Polkinghorne A. (2018). Characterization of the *in vitro chlamydia pecorum* response to gamma interferon. Infect. Immun. 86 (4), e00714–17. doi: 10.1128/iai.00714-17 29358337 PMC5865032

[B65] Jelocnik M. Bachmann N. L. Kaltenboeck B. Waugh C. Woolford L. Speight K. N.. (2015). Genetic diversity in the plasticity zone and the presence of the chlamydial plasmid differentiates *Chlamydia pecorum* strains from pigs, sheep, cattle, and koalas. BMC Genom. 16, 893. doi: 10.1186/s12864-015-2053-8 PMC4632680 26531162

[B66] Jelocnik M. Polkinghorne A. Pannekoek Y. (2019). Multilocus sequence typing (MLST) of *Chlamydiales* . Methods Mol. Biol. 2042, 69–86. doi: 10.1007/978-1-4939-9694-0_7 31385271

[B67] Jenkins C. Jelocnik M. Micallef M. L. Galea F. Taylor-Brown A. Bogema D. R.. (2018). An epizootic of *Chlamydia psittaci* equine reproductive loss associated with suspected spillover from native Australian parrots. Emerg. Microbes Infect. 7 (1), 88. doi: 10.1038/s41426-018-0089-y 29765033 PMC5953950

[B68] Jolley K. A. Bray J. E. Maiden M. C. J. (2018). Open-access bacterial population genomics: BIGSdb software, the PubMLST.org website and their applications. Wellcome Open Res. 3, 124. doi: 10.12688/wellcomeopenres.14826.1 30345391 PMC6192448

[B69] Jones C. A. Hadfield J. Thomson N. R. Cleary D. W. Marsh P. Clarke I. N.. (2020). The nature and extent of plasmid variation in *Chlamydia trachomatis* . Microorganisms 8 (3), 373. doi: 10.3390/microorganisms8030373 32155798 PMC7143637

[B70] Joseph S. J. Didelot X. Rothschild J. de Vries H. J. Morré S. A. Read T. D.. (2012). Population genomics of *Chlamydia trachomatis*: insights on drift, selection, recombination, and population structure. Mol. Biol. Evol. 29 (12), 3933–3946. doi: 10.1093/molbev/mss198 22891032 PMC3494276

[B71] Joseph S. J. Marti H. Didelot X. Castillo-Ramirez S. Read T. D. Dean D. (2015). *chlamydiaceae* genomics reveals interspecies admixture and the recent evolution of *Chlamydia abortus* infecting lower mammalian species and humans. Genome Biol. Evol. 7 (11), 3070–3084. doi: 10.1093/gbe/evv201 26507799 PMC4994753

[B72] Joseph S. J. Marti H. Didelot X. Read T. D. Dean D. (2016). Tetracycline selective pressure and homologous recombination shape the evolution of *Chlamydia suis*: a recently identified zoonotic pathogen. Genome Biol. Evol. 8 (8), 2613–2623. doi: 10.1093/gbe/evw182 27576537 PMC5010913

[B73] Karunakaran K. P. Rey-Ladino J. Stoynov N. Berg K. Shen C. Jiang X.. (2008). Immunoproteomic discovery of novel T cell antigens from the obligate intracellular pathogen *Chlamydia* . J. Immunol. 180 (4), 2459–2465. doi: 10.4049/jimmunol.180.4.2459 18250455

[B74] Kuchina A. Brettner L. M. Paleologu L. Roco C. M. Rosenberg A. B. Carignano A.. (2021). Microbial single-cell RNA sequencing by split-pool barcoding. Science 371 (6531), eaba5257. doi: 10.1126/science.aba5257 33335020 PMC8269303

[B75] Lamy-Besnier Q. Koszul R. Debarbieux L. Marbouty M. (2021). Closed and high-quality bacterial genome sequences of the oligo-mouse-microbiota community. Microbiol. Resour. Announc. 10 (17), e01396–20. doi: 10.1128/mra.01396-20 33927045 PMC8086220

[B76] Liang P. Rosas-Lemus M. Patel D. Fang X. Tuz K. Juárez O. (2018). Dynamic energy dependency of *Chlamydia trachomatis* on host cell metabolism during intracellular growth: role of sodium-based energetics in chlamydial ATP generation. J. Biol. Chem. 293 (2), 510–522. doi: 10.1074/jbc.M117.797209 29123027 PMC5767857

[B77] Liu S.-Y. Li K.-P. Hsieh M.-K. Chang P.-C. Shien J.-H. Ou S.-C. (2019). Prevalence and genotyping of *Chlamydia psittaci* from domestic waterfowl, companion birds, and wild birds in Taiwan. Vector-Borne Zoonotic Dis. 19 (9), 666–673. doi: 10.1089/vbz.2018.2403 30855216

[B78] Livingstone M. Caspe S. G. Longbottom D. (2021). Complete genome sequence of *Chlamydia abortus* MRI-10/19, isolated from a sheep vaccinated with the commercial live *C. abortus* 1B vaccine strain. Microbiol. Resour. Announc. 10 (18), e00203–21. doi: 10.1128/mra.00203-21 33958416 PMC8103861

[B79] Longbottom D. Livingstone M. Aitchison K. D. Imrie L. Manson E. Wheelhouse N.. (2019). Proteomic characterisation of the *Chlamydia abortus* outer membrane complex (COMC) using combined rapid monolithic column liquid chromatography and fast MS/MS scanning. PloS One 14 (10), e0224070. doi: 10.1371/journal.pone.0224070 31647835 PMC6812762

[B80] Longbottom D. Livingstone M. Ribeca P. Beeckman D. S. A. van der Ende A. Pannekoek Y.. (2021). Whole genome *de novo* sequencing and comparative genomic analyses suggests that *Chlamydia psittaci* strain 84/2334 should be reclassified as *Chlamydia abortus* species. BMC Genom. 22 (1), 159. doi: 10.1186/s12864-021-07477-6 PMC7937271 33676404

[B81] Lutter E. I. Martens C. Hackstadt T. (2012). Evolution and conservation of predicted inclusion membrane proteins in *Chlamydiae* . Comp. Funct. Genomics 2012, 362104. doi: 10.1155/2012/362104 22454599 PMC3290821

[B82] Marschall M. T. Simnacher U. Walther P. Essig A. Hagemann J. B. (2020). The putative type iii secreted *Chlamydia abortus* virulence-associated protein CAB063 targets lamin and induces apoptosis. Front. Microbiol. 11. doi: 10.3389/fmicb.2020.01059 PMC7261910 32523581

[B83] Marti H. Bommana S. Read T. D. Pesch T. Prähauser B. Dean D.. (2021). Generation of tetracycline and rifamycin resistant *Chlamydia suis* recombinants. Front. Microbiol. 12. doi: 10.3389/fmicb.2021.630293 PMC8278220 34276577

[B84] Marti H. Kim H. Joseph S. J. Dojiri S. Read T. D. Dean D. (2017). *Tet*(C) gene transfer between *Chlamydia suis* strains occurs by homologous recombination after co-infection: implications for spread of tetracycline-resistance among *Chlamydiaceae* . Front. Microbiol. 8. doi: 10.3389/fmicb.2017.00156 PMC5293829 28223970

[B85] Marti H. Suchland R. J. Rockey D. D. (2022). The impact of lateral gene transfer in *Chlamydia* . Front. Cell Infect. Microbiol. 12. doi: 10.3389/fcimb.2022.861899 PMC8936141 35321311

[B86] McClarty G. Qin B. (1993). Pyrimidine metabolism by intracellular *Chlamydia psittaci* . J. Bacteriol 175 (15), 4652–4661. doi: 10.1128/jb.175.15.4652-4661.1993 8335624 PMC204916

[B87] McQueen B. E. Kiatthanapaiboon A. Fulcher M. L. Lam M. Patton K. Powell E.. (2020). Human fallopian tube epithelial cell culture model to study host responses to *Chlamydia trachomatis* infection. Infect. Immun. 88 (9), e00105–20. doi: 10.1128/iai.00105-20 32601108 PMC7440757

[B88] Mojica S. A. Hovis K. M. Frieman M. B. Tran B. Hsia R.-c. Ravel J.. (2015). SINC, a type III secreted protein of *Chlamydia psittaci*, targets the inner nuclear membrane of infected cells and uninfected neighbors. Mol. Biol. Cell 26 (10), 1918–1934. doi: 10.1091/mbc.E14-11-1530 25788290 PMC4436835

[B89] Mölleken K. Schmidt E. Hegemann J. H. (2010). Members of the pmp protein family of *Chlamydia pneumoniae* mediate adhesion to human cells *via* short repetitive peptide motifs. Mol. Microbiol. 78 (4), 1004–1017. doi: 10.1111/j.1365-2958.2010.07386.x 21062373 PMC2997323

[B90] Moran N. A. (2002). Microbial minimalism: genome reduction in bacterial pathogens. Cell 108 (5), 583–586. doi: 10.1016/s0092-8674(02)00665-7 11893328

[B91] Mueller K. E. Plano G. V. Fields K. A. (2014). New frontiers in type III secretion biology: the *Chlamydia* perspective. Infect. Immun. 82 (1), 2–9. doi: 10.1128/IAI.00917-13 24126521 PMC3911841

[B92] Nogueira A. T. Braun K. M. Carabeo R. A. (2017). Characterization of the growth of *Chlamydia trachomatis* in *in vitro*-generated stratified epithelium. Front. Cell Infect. Microbiol. 7. doi: 10.3389/fcimb.2017.00438 PMC5641298 29067282

[B93] Nunes A. Borrego M. J. Gomes J. P. (2013). Genomic features beyond *Chlamydia trachomatis* phenotypes: what do we think we know? Infect. Genet. Evol. 16, 392–400. doi: 10.1016/j.meegid.2013.03.018 23523596

[B94] Nunes A. Borrego M. J. Nunes B. Florindo C. Gomes J. P. (2009). Evolutionary dynamics of *omp*A, the gene encoding the *Chlamydia trachomatis* key antigen. J. Bacteriol 191 (23), 7182–7192. doi: 10.1128/JB.00895-09 19783629 PMC2786549

[B95] Nunes A. Gomes J. P. (2014). Evolution, phylogeny, and molecular epidemiology of *Chlamydia* . Infect. Genet. Evol. 23, 49–64. doi: 10.1016/j.meegid.2014.01.029 24509351

[B96] Nunes A. Gomes J. P. Karunakaran K. P. Brunham R. C. (2015). Bioinformatic analysis of *Chlamydia trachomatis* polymorphic membrane proteins PmpE, PmpF, PmpG and PmpH as potential vaccine antigens. PloS One 10 (7), e0131695. doi: 10.1371/journal.pone.0131695 26131720 PMC4488443

[B97] Olsen A. W. Rosenkrands I. Holland M. J. Andersen P. Follmann F. (2021). A *Chlamydia trachomatis* VD1-MOMP vaccine elicits cross-neutralizing and protective antibodies against C/C-related complex serovars. NPJ Vaccines 6 (1), 58. doi: 10.1038/s41541-021-00312-9 33875654 PMC8055873

[B98] Østergaard O. Follmann F. Olsen A. W. Heegaard N. H. Andersen P. Rosenkrands I. (2016). Quantitative protein profiling of *Chlamydia trachomatis* growth forms reveals defense strategies against tryptophan starvation. Mol. Cell Proteom. 15 (12), 3540–3550. doi: 10.1074/mcp.M116.061986 PMC5141270 27784728

[B99] Panzetta M. E. Valdivia R. H. Saka H. A. (2018). Chlamydia persistence: a survival strategy to evade antimicrobial effects *in-vitro* and *in-vivo* . Front. Microbiol. 9. doi: 10.3389/fmicb.2018.03101 PMC6299033 30619180

[B100] Patiño L. H. Camargo M. Muñoz M. Ríos-Chaparro D. I. Patarroyo M. A. Ramírez J. D. (2018). Unveiling the multilocus sequence typing (MLST) schemes and core genome phylogenies for genotyping *Chlamydia trachomatis* . Front. Microbiol. 9. doi: 10.3389/fmicb.2018.01854 PMC6113918 30186244

[B101] Pawlikowska-Warych M. Śliwa-Dominiak J. Deptuła W. (2015). Chlamydial plasmids and bacteriophages. Acta Biochim. Pol. 62 (1), 1–6. doi: 10.18388/abp.2014_764 25654356

[B102] Peters J. Wilson D. P. Myers G. Timms P. Bavoil P. M. (2007). Type III secretion à la *Chlamydia* . Trends Microbiol. 15 (6), 241–251. doi: 10.1016/j.tim.2007.04.005 17482820

[B103] Pillonel T. Bertelli C. Greub G. (2018). Environmental metagenomic assemblies reveal seven new highly divergent chlamydial lineages and hallmarks of a conserved intracellular lifestyle. Front. Microbiol. 9. doi: 10.3389/fmicb.2018.00079 PMC5826181 29515524

[B104] Pillonel T. Tagini F. Bertelli C. Greub G. (2020). ChlamDB: a comparative genomics database of the phylum *Chlamydiae* and other members of the *Planctomycetes*-*Verrucomicrobiae*-*Chlamydiae* superphylum. Nucleic Acids Res. 48 (D1), D526–d534. doi: 10.1093/nar/gkz924 31665454 PMC7145651

[B105] Piñeiro L. Bernal S. Bordes A. Palomares J. C. Gilarranz R. von Wichmann M. A.. (2014). Minimum spread of the new Swedish variant of *Chlamydia trachomatis* and distribution of *C. trachomatis omp*A genotypes in three geographically distant areas of Spain 2011-2012. Infection 42 (5), 905–912. doi: 10.1007/s15010-014-0665-6 25056129

[B108] Pokorzynski N. D. Alla M. R. Carabeo R. A. (2022). Host cell amplification of nutritional stress contributes to persistence in *Chlamydia trachomatis* . mBio 13 (6), e0271922. doi: 10.1128/mbio.02719-22 36377897 PMC9765610

[B106] Pokorzynski N. D. Brinkworth A. J. Carabeo R. (2019). A bipartite iron-dependent transcriptional regulation of the tryptophan salvage pathway in *Chlamydia trachomatis* . Elife 8, e42295. doi: 10.7554/eLife.42295 30938288 PMC6504234

[B107] Pokorzynski N. D. Hatch N. D. Ouellette S. P. Carabeo R. (2020). The iron-dependent repressor YtgR is a tryptophan-dependent attenuator of the *trpRBA* operon in *Chlamydia trachomatis* . Nat. Commun. 11, 6430. doi: 10.1038/s41467-020-20181-5 33353937 PMC7755916

[B109] Porcella S. F. Carlson J. H. Sturdevant D. E. Sturdevant G. L. Kanakabandi K. Virtaneva K.. (2015). Transcriptional profiling of human epithelial cells infected with plasmid-bearing and plasmid-deficient *Chlamydia trachomatis* . Infect. Immun. 83 (2), 534–543. doi: 10.1128/iai.02764-14 25404022 PMC4294249

[B110] Quigley B. L. Timms P. (2021). The koala immune response to chlamydial infection and vaccine development-advancing our immunological understanding. Anim. (Basel) 11 (2), 380. doi: 10.3390/ani11020380 PMC7913230 33546104

[B111] Rajaram K. Giebel A. M. Toh E. Hu S. Newman J. H. Morrison S. G.. (2015). Mutational analysis of the *Chlamydia muridarum* plasticity zone. Infect. Immun. 83 (7), 2870–2881. doi: 10.1128/iai.00106-15 25939505 PMC4468545

[B112] Rajeeve K. Vollmuth N. Janaki-Raman S. Wulff T. F. Baluapuri A. Dejure F. R.. (2020). Reprogramming of host glutamine metabolism during *Chlamydia trachomatis* infection and its key role in peptidoglycan synthesis. Nat. Microbiol. 5 (11), 1390–1402. doi: 10.1038/s41564-020-0762-5 32747796

[B113] Rawre J. Dhawan B. Khanna N. Sreenivas V. Broor S. Chaudhry R. (2019). Distribution of *Chlamydia trachomatis omp*A genotypes in patients attending a sexually transmitted disease outpatient clinic in new Delhi, India. Indian J. Med. Res. 149 (5), 662–670. doi: 10.4103/ijmr.IJMR_1171_17 31417035 PMC6702700

[B114] Read T. D. Joseph S. J. Didelot X. Liang B. Patel L. Dean D. (2013). Comparative analysis of *Chlamydia psittaci* genomes reveals the recent emergence of a pathogenic lineage with a broad host range. mBio 4 (2), e00604–e00612. doi: 10.1128/mBio.00604-12 PMC3622922 23532978

[B115] Robbins A. Hanger J. Jelocnik M. Quigley B. L. Timms P. (2019). Longitudinal study of wild koalas (*Phascolarctos cinereus*) reveals chlamydial disease progression in two thirds of infected animals. Sci. Rep. 9 (1), 13194. doi: 10.1038/s41598-019-49382-9 31519969 PMC6744427

[B116] Rockey D. D. (2011). Unraveling the basic biology and clinical significance of the chlamydial plasmid. J. Exp. Med. 208 (11), 2159–2162. doi: 10.1084/jem.20112088 22025500 PMC3201210

[B117] Rother M. Gonzalez E. Teixeira da Costa A. R. Wask L. Gravenstein I. Pardo M.. (2018). Combined human genome-wide rnai and metabolite analyses identify IMPDH as a host-directed target against *Chlamydia* infection. Cell Host Microbe 23 (5), 661–671.e668. doi: 10.1016/j.chom.2018.04.002 29706504

[B118] Roulis E. Bachmann N. Humphrys M. Myers G. Huston W. Polkinghorne A.. (2015a). Phylogenetic analysis of human *Chlamydia pneumoniae* strains reveals a distinct Australian indigenous clade that predates European exploration of the continent. BMC Genom. 16, 1094–1094. doi: 10.1186/s12864-015-2281-y PMC4687280 26694618

[B119] Roulis E. Bachmann N. L. Myers G. S. Huston W. Summersgill J. Hudson A.. (2015b). Comparative genomic analysis of human *Chlamydia pneumoniae* isolates from respiratory, brain and cardiac tissues. Genomics 106 (6), 373–383. doi: 10.1016/j.ygeno.2015.09.008 26420648

[B120] Saka H. A. Valdivia R. H. (2010). Acquisition of nutrients by *Chlamydiae*: unique challenges of living in an intracellular compartment. Curr. Opin. Microbiol. 13 (1), 4–10. doi: 10.1016/j.mib.2009.11.002 20006538 PMC3202608

[B122] Seth-Smith H. M. B. Bénard A. Bruisten S. M. Versteeg B. Herrmann B. Kok J.. (2021). Ongoing evolution of *Chlamydia trachomatis* lymphogranuloma venereum: exploring the genomic diversity of circulating strains. Microb. Genom 7 (6), 599. doi: 10.1099/mgen.0.000599 PMC8461462 34184981

[B123] Seth-Smith H. M. B. Busó L. S. Livingstone M. Sait M. Harris S. R. Aitchison K. D.. (2017b). European *Chlamydia abortus* livestock isolate genomes reveal unusual stability and limited diversity, reflected in geographical signatures. BMC Genom 18 (1), 344. doi: 10.1186/s12864-017-3657-y PMC5415952 28472926

[B124] Seth-Smith H. M. B. Harris S. R. Persson K. Marsh P. Barron A. Bignell A.. (2009). Co-Evolution of genomes and plasmids within *Chlamydia trachomatis* and the emergence in Sweden of a new variant strain. BMC Genom. 10, 239–239. doi: 10.1186/1471-2164-10-239 PMC2693142 19460133

[B121] Seth-Smith H. M. Wanninger S. Bachmann N. Marti H. Qi W. Donati M.. (2017a). The *Chlamydia suis* genome exhibits high levels of diversity, plasticity, and mobile antibiotic resistance: comparative genomics of a recent livestock cohort shows influence of treatment regimes. Genome Biol. Evol. 9 (3), 750–760. doi: 10.1093/gbe/evx043 28338777 PMC5381551

[B125] Sigalova O. M. Chaplin A. V. Bochkareva O. O. Shelyakin P. V. Filaretov V. A. Akkuratov E. E.. (2019). *Chlamydia* pan-genomic analysis reveals balance between host adaptation and selective pressure to genome reduction. BMC Genom 20 (1), 710. doi: 10.1186/s12864-019-6059-5 PMC6740158 31510914

[B126] Sigar I. M. Schripsema J. H. Wang Y. Clarke I. N. Cutcliffe L. T. Seth-Smith H. M.. (2014). Plasmid deficiency in urogenital isolates of *Chlamydia trachomatis* reduces infectivity and virulence in a mouse model. Pathog. Dis. 70 (1), 61–69. doi: 10.1111/2049-632x.12086 24022847 PMC4300952

[B127] Skipp P. J. Hughes C. McKenna T. Edwards R. Langridge J. Thomson N. R.. (2016). Quantitative proteomics of the infectious and replicative forms of *Chlamydia trachomatis* . PloS One 11 (2), e0149011. doi: 10.1371/journal.pone.0149011 26871455 PMC4752267

[B128] Somboonna N. Ziklo N. Ferrin T. E. Hyuk Suh J. Dean D. (2019). Clinical persistence of *Chlamydia trachomatis* sexually transmitted strains involves novel mutations in the functional αββα tetramer of the tryptophan synthase operon. mBio 10 (4), e01464–19. doi: 10.1128/mBio.01464-19 31311884 PMC6635532

[B129] Song L. Carlson J. H. Whitmire W. M. Kari L. Virtaneva K. Sturdevant D. E.. (2013). *Chlamydia trachomatis* plasmid-encoded Pgp4 is a transcriptional regulator of virulence-associated genes. Infect. Immun. 81 (3), 636–644. doi: 10.1128/iai.01305-12 23319558 PMC3584862

[B130] Suchland R. J. Carrell S. J. Wang Y. Hybiske K. Kim D. B. Dimond Z. E.. (2019). Chromosomal recombination targets in *Chlamydia* interspecies lateral gene transfer. J. Bacteriol 201 (23), 4604–4611. doi: 10.1128/jb.00365-19 PMC6832074 31501285

[B131] Suchland R. J. Sandoz K. M. Jeffrey B. M. Stamm W. E. Rockey D. D. (2009). Horizontal transfer of tetracycline resistance among *Chlamydia* spp. *in vitro* . Antimicrob. Agents Chemother. 53 (11), 4604–4611. doi: 10.1128/aac.00477-09 19687238 PMC2772348

[B132] Sun G. Pal S. Sarcon A. K. Kim S. Sugawara E. Nikaido H.. (2007). Structural and functional analyses of the major outer membrane protein of *Chlamydia trachomatis* . J. Bacteriol. 189 (17), 6222–6235. doi: 10.1128/JB.00552-07 17601785 PMC1951919

[B133] Szabo K. V. O’Neill C. E. Clarke I. N. (2020). Diversity in chlamydial plasmids. PloS One 15 (5), e0233298. doi: 10.1371/journal.pone.0233298 32469898 PMC7259575

[B134] Taylor-Brown A. Madden D. Polkinghorne A. (2018). Culture-independent approaches to chlamydial genomics. Microb. Genom. 4 (2), e000145. doi: 10.1099/mgen.0.000145 29310749 PMC5857372

[B135] Tifrea D. F. Pal S. de la Maza L. M. (2020). A recombinant *Chlamydia trachomatis* MOMP vaccine elicits cross-serogroup protection in mice against vaginal shedding and infertility. J. Infect. Dis. 221 (2), 191–200. doi: 10.1093/infdis/jiz438 31504647 PMC6935996

[B136] Tourancheau A. Mead E. A. Zhang X. S. Fang G. (2021). Discovering multiple types of DNA methylation from bacteria and microbiome using nanopore sequencing. Nat. Methods 18 (5), 491–498. doi: 10.1038/s41592-021-01109-3 33820988 PMC8107137

[B137] Turman B. J. Alzhanov D. Nagarajan U. M. Darville T. O'Connell C. M. (2023). Virulence protein Pgp3 is insufficient to mediate plasmid-dependent infectivity of *Chlamydia trachomatis* . Infect. Immun. 91 (2), e0039222. doi: 10.1128/iai.00392-22 36722979 PMC9933628

[B138] Valdivia R. H. (2008). *Chlamydia* effector proteins and new insights into chlamydial cellular microbiology. Curr. Opin. Microbiol. 11 (1), 53–59. doi: 10.1016/j.mib.2008.01.003 18299248

[B139] Vasilevsky S. Stojanov M. Greub G. Baud D. (2016). Chlamydial polymorphic membrane proteins: regulation, function and potential vaccine candidates. Virulence 7 (1), 11–22. doi: 10.1080/21505594.2015.1111509 26580416 PMC4871649

[B140] Versteeg B. Bruisten S. M. Pannekoek Y. Jolley K. A. Maiden M. C. J. van der Ende A.. (2018a). Genomic analyses of the *Chlamydia trachomatis* core genome show an association between chromosomal genome, plasmid type and disease. BMC Genom 19 (1), 130. doi: 10.1186/s12864-018-4522-3 PMC5810182 29426279

[B141] Versteeg B. van den Broek L. J. Bruisten S. M. Mullender M. de Vries H. J. C. Gibbs S. (2018b). An organotypic reconstructed human urethra to study *Chlamydia trachomatis* infection. Tissue Eng. Part A 24 (21-22), 1663–1671. doi: 10.1089/ten.TEA.2017.0511 29792385

[B142] Voigt A. Schöfl G. Saluz H. P. (2012). The *Chlamydia psittaci* genome: a comparative analysis of intracellular pathogens. PloS One 7 (4), e35097. doi: 10.1371/journal.pone.0035097 22506068 PMC3323650

[B143] Vorimore F. Aaziz R. de Barbeyrac B. Peuchant O. Szymańska-Czerwińska M. Herrmann B.. (2021a). A new SNP-based genotyping method for *C. psittaci*: application to field samples for quick identification. Microorganisms 9 (3), 625. doi: 10.3390/microorganisms9030625 33803059 PMC8002925

[B144] Vorimore F. Hölzer M. Liebler-Tenorio E. M. Barf L. M. Delannoy S. Vittecoq M.. (2021b). Evidence for the existence of a new genus *Chlamydiifrater* gen. nov. inside the family *Chlamydiaceae* with two new species isolated from flamingo (*Phoenicopterus roseus*): chlamydiifrater phoenicopteri sp. nov. and *Chlamydiifrater volucris* sp. nov. Syst. Appl. Microbiol. 44 (4), 126200. doi: 10.1016/j.syapm.2021.126200 34298369

[B145] Wen Z. Boddicker M. A. Kaufhold R. M. Khandelwal P. Durr E. Qiu P.. (2016). Recombinant expression of *Chlamydia trachomatis* major outer membrane protein in *E. coli* outer membrane as a substrate for vaccine research. BMC Microbiol. 16 (1), 165. doi: 10.1186/s12866-016-0787-3 27464881 PMC4963994

[B146] White R. T. Anstey S. I. Kasimov V. Jenkins C. Devlin J. El-Hage C.. (2022). One clone to rule them all: culture-independent genomics of *Chlamydia psittaci* from equine and avian hosts in Australia. Microb. Genom. 8 (10), mgen000888. doi: 10.1099/mgen.0.000888 36269227 PMC9676050

[B147] White R. T. Jelocnik M. Klukowski N. Haque M. H. Sarker S. (2023). The first genomic insight into *Chlamydia psittaci* sequence type (ST)24 from a healthy captive psittacine host in Australia demonstrates evolutionary proximity with strains from psittacine, human, and equine hosts. Vet. Microbiol. 280, 109704. doi: 10.1016/j.vetmic.2023.109704 36840991

[B148] White R. T. Legione A. R. Taylor-Brown A. Fernandez C. M. Higgins D. P. Timms P.. (2021). Completing the genome sequence of *Chlamydia pecorum* strains MC/MarsBar and DBDeUG: new insights into this enigmatic koala (*Phascolarctos cinereus*) pathogen. Pathogens 10 (12), 1543. doi: 10.3390/pathogens10121543 34959498 PMC8703710

[B149] Wolff B. J. Morrison S. S. Pesti D. Ganakammal S. R. Srinivasamoorthy G. Changayil S.. (2015). *Chlamydia psittaci* comparative genomics reveals intraspecies variations in the putative outer membrane and type III secretion system genes. Microbiology 161 (7), 1378–1391. doi: 10.1099/mic.0.000097 25887617 PMC4635502

[B150] Wood H. Roshick C. McClarty G. (2004). Tryptophan recycling is responsible for the interferon-gamma resistance of chlamydia psittaci GPIC in indoleamine dioxygenase-expressing host cells. Mol. Microbiol. 52 (3), 903–916. doi: 10.1111/j.1365-2958.2004.04029.x 15101993

[B151] Yang C. Kari L. Lei L. Carlson J. H. Ma L. Couch C. E.. (2020). *Chlamydia trachomatis* plasmid gene protein 3 is essential for the establishment of persistent infection and associated immunopathology. mBio 11 (4), e01902–20. doi: 10.1128/mBio.01902-20 32817110 PMC7439461

[B152] Yang M. Rajeeve K. Rudel T. Dandekar T. (2019). Comprehensive flux modeling of *Chlamydia trachomatis* proteome and qRT-PCR data indicate biphasic metabolic differences between elementary bodies and reticulate bodies during infection. Front. Microbiol. 10. doi: 10.3389/fmicb.2019.02350 PMC6803457 31681215

[B153] Zadora P. K. Chumduri C. Imami K. Berger H. Mi Y. Selbach M.. (2019). Integrated phosphoproteome and transcriptome analysis reveals *Chlamydia*-induced epithelial-to-mesenchymal transition in host cells. Cell Rep. 26 (5), 1286–1302.e1288. doi: 10.1016/j.celrep.2019.01.006 30699355

[B154] Zaręba-Marchewka K. Szymańska-Czerwińska M. Livingstone M. Longbottom D. Niemczuk K. (2021). Whole genome sequencing and comparative genome analyses of *Chlamydia abortus* strains of avian origin suggests that *Chlamydia abortus* species should be expanded to include avian and mammalian subgroups. Pathogens 10 (11), 461–467. doi: 10.3390/pathogens10111405 34832561 PMC8623937

[B155] Zaręba-Marchewka K. Szymańska-Czerwińska M. Niemczuk K. (2020). *Chlamydiae* - what's new? J. Vet. Res. 64 (4), 461–467. doi: 10.2478/jvetres-2020-0077 33367133 PMC7734683

[B156] Zhao Y. Wang J. Chen J. Zhang X. Guo M. Yu G. (2020). A literature review of gene function prediction by modeling gene ontology. Front. Genet. 11. doi: 10.3389/fgene.2020.00400 PMC7193026 32391061

[B157] Zhong G. (2017). Chlamydial plasmid-dependent pathogenicity. Trends Microbiol. 25 (2), 141–152. doi: 10.1016/j.tim.2016.09.006 27712952 PMC5272858

[B158] Zhou L. Lopez Rodas A. Llangarí L. M. Romero Sandoval N. Cooper P. Sadiq S. T. (2022). Single gene targeted nanopore sequencing enables simultaneous identification and antimicrobial resistance detection of sexually transmitted infections. PloS One 17 (1), e0262242. doi: 10.1371/journal.pone.0262242 35061780 PMC8782522

[B159] Ziklo N. Huston W. M. Taing K. Katouli M. Timms P. (2016). *In vitro* rescue of genital strains of *Chlamydia trachomatis* from interferon-γ and tryptophan depletion with indole-positive, but not indole-negative *Prevotella* spp. BMC Microbiol. 16 (1), 286. doi: 10.1186/s12866-016-0903-4 27914477 PMC5135834

